# Effects of Glucosinolate-Derived Isothiocyanates on Fungi: A Comprehensive Review on Direct Effects, Mechanisms, Structure-Activity Relationship Data and Possible Agricultural Applications

**DOI:** 10.3390/jof7070539

**Published:** 2021-07-06

**Authors:** Tamás Plaszkó, Zsolt Szűcs, Gábor Vasas, Sándor Gonda

**Affiliations:** 1 Department of Botany, Division of Pharmacognosy, University of Debrecen, Egyetem tér 1, 4032 Debrecen, Hungary; plaszko.tamas@tutamail.com (T.P.); szucs.zsolt@science.unideb.hu (Z.S.); vasas.gabor@science.unideb.hu (G.V.); 2 Doctoral School of Pharmaceutical Sciences, University of Debrecen, 4032 Debrecen, Hungary; 3 Healthcare Industry Institute, University of Debrecen, 4032 Debrecen, Hungary

**Keywords:** antifungal natural products, biofumigation, *Cruciferae*, fungi, glucosinolates, isothiocyanates, QSAR, synergistic activity, VOC, grain storage, crop protection

## Abstract

Plants heavily rely on chemical defense systems against a variety of stressors. The glucosinolates in the *Brassicaceae* and some allies are the core molecules of one of the most researched such pathways. These natural products are enzymatically converted into isothiocyanates (ITCs) and occasionally other defensive volatile organic constituents (VOCs) upon fungal challenge or tissue disruption to protect the host against the stressor. The current review provides a comprehensive insight on the effects of the isothiocyanates on fungi, including, but not limited to mycorrhizal fungi and pathogens of *Brassicaceae*. In the review, our current knowledge on the following topics are summarized: direct antifungal activity and the proposed mechanisms of antifungal action, QSAR (quantitative structure-activity relationships), synergistic activity of ITCs with other agents, effects of ITCs on soil microbial composition and allelopathic activity. A detailed insight into the possible applications is also provided: the literature of biofumigation studies, inhibition of post-harvest pathogenesis and protection of various products including grains and fruits is also reviewed herein.

## 1. Introduction

Plants are sessile organisms, that lack motile immune system elements. Therefore, to cope with abiotic and biotic stresses, they rapidly adapt their metabolism and deploy various so-called specialized metabolites to help them defend against various stressors [1]. These specialized compounds are biosynthesized in a fine-tuned manner to defend against pathogens.

**Figure 1:**
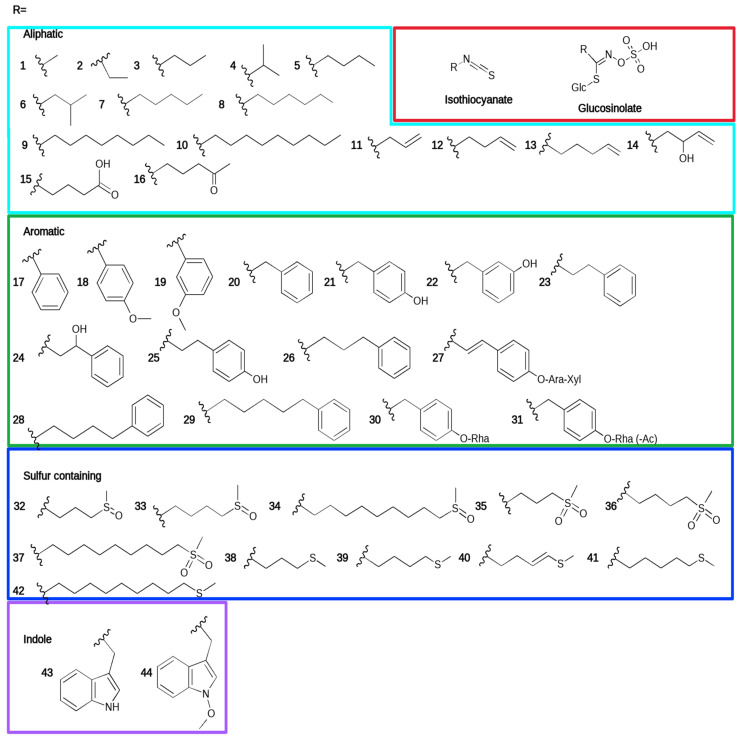
Glucosinolates and isothiocyanates mentioned in the current review. The classification is based on [5]. If available, the trivial names for glucosinolates are mentioned in the absence of trivial names, the GSL name is formed from the side chain name. Abbreviations: Ac—acetyl-group, Ara—arabinoside; Glc—glucoside; ITC—isothiocyanate; Rha—rhamnoside; Xyl—xyloside. 1. methyl ITC/Glucocapparin; 2. ethyl ITC/Glucolepidiin; 3. propyl ITC; 4. i-propyl ITC/Glucoputranjivin; 5. butyl ITC; 6. iso-butyl ITC; 7. pentyl ITC; 8. hexyl ITC; 9. octyl ITC; 10. nonyl ITC; 11. 2-propenyl ITC (allyl ITC)/sinigrin; 12. 3-butenyl ITC/Gluconapin; 13. 4-pentenyl ITC/Glucobrassicanapin; 14. 2-hydroxy-3-butenyl ITC/Progoitrin; 15. 4-isothiocyanatobutanoic acid; 16. 4-oxopentyl ITC; 17. phenyl ITC; 18. 4-methoxybenzyl ITC/Glucoaubrietin; 19. 3-methoxybenzyl ITC/Glucolimnanthin; 20. benzyl ITC/Glucotropaeolin; 21. 4-hydroxybenzyl ITC/sinalbin; 22. 3-hydroxybenzyl ITC/Glucolepigramin; 23. phenethyl ITC/Gluconasturtiin; 24. hydroxyphenethyl ITC/Glucobarbarin, 25. 4-hydroxyphenethyl ITC; 26. 3-phenylpropyl ITC; 27. sinapigladioside; 28. 4-phenylbutyl ITC; 29. 5-phenylpentyl ITC; 30. 4-(a-L-rhamnosyloxy)-benzyl ITC; 31. 4-(4′-O-acetyl-a-L-rhamnosyloxy)-benzyl ITC; 32. 3-(methylsulfinyl)propyl ITC; 33. 4-(methylsulfinyl)butyl ITC (sulforaphane)/glucoraphanin; 34. 9-(methylsulfinyl)nonyl ITC/Glucoarabin; 35. 3-(methylsulfonyl)propyl ITC; 36. 4-(methylsulfonyl)butyl ITC; 37. 9-(methylsulfonyl)nonyl ITC; 38. 3-(methylsulfanyl)propyl ITC (iberverin)/Glucoiberverin; 39. 4-(methylthio)butyl ITC (erucin)/Glucoerucin; 40. 4-methylsulfinyl-3-butenyl ITC (sulforaphene)/Glucoraphanin; 41. 5-(methylthio)pentyl ITC (Berteroin); 42. 9-(methylthio)nonyl ITC; 43. indol-3-ylmethyl ITC/Glucobrassicin; 44. 1-methoxyindol-3-ylmethyl ITC/Neoglucobrassicin.

One of the most well-studied chemical defense systems of plants is the glucosinolate (GSL) pathway, found in the plants of the order Brassicales [2,3]. In contrast to many other specialized metabolites such as phenolic compounds, these natural products are not bioactive themselves, but rely on an activating enzyme that produces the actual bioactive agents from them upon demand [4]. In philosophy, this is similar to the case of cyanogenic glycosides [1]. The compounds originate from amino acids, and show a considerable variability in side chain chemistry [5]. The isothiocyanates (ITCs) mentioned in the study are plotted in Figure 1, along with their precursor GSLs.

Though the inability to biosynthesize GSLs clearly results in an increase of sensitivity towards many types of fungal pathogens, native GSLs themselves show no direct antifungal activity, as shown in the literature, for example in [6,7]. The antifungal defense machinery is therefore thought to rely on decomposition products from GSLs. Currently, in planta GSL decomposition is not fully understood and therefore the list of actual antifungal agents is incomplete: recent papers concluded that unknown decomposition products may contribute to in vivo antifungal effects [8,9,10], but data show that, without doubt, the most potent antifungal GSL decomposition products are ITCs.

**Figure 2:**
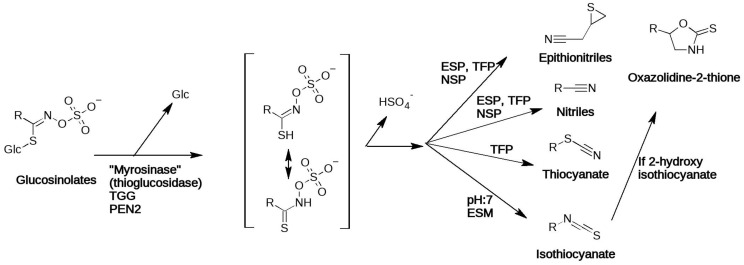
Deglucosylation and subsequent spontaneous rearrangement of glucosinolates into various volatile natural products. Compiled from data in [4,13]. Abbreviations: ESM—epithiospecifier modifier protein; ESP—epithionitrile specifier protein; Glc—glucose; NSP—nitrile specifier protein; PEN2—atypical myrosinase; TGG—beta-thioglucoside glucohydrolase (myrosinase); TFP—thiocyanate-forming protein.

Production of bioactive volatiles from GSLs starts with the enzymatic hydrolysis of the GSL thioglucosidic bond (Figure 2). Deglucosylation is done by myrosinase (beta-thioglucoside glucohydrolase, TGG in *A. thaliana*) during plant tissue damage, or alternative (“non-typical”) myrosinases in intact tissues. The latter include PEN2 and likely other, currently unknown enzymes as well. Myrosinases are members of glycoside hydrolase family I, just like other glucosidases playing a role in plant defense [4]. Plants usually express several isoforms, different in spatial, temporal distribution and enzymatic properties. Though the enzymatic activity towards different GSLs is usually different [11], the myrosinase enzymes typically accept all GSLs as substrates [12]. The resulting unstable GSL aglycon (Figure 2) thiohydroximate-O-sulfate then subsequently rearranges to form various volatile decomposition products: the default rearrangement products are ITCs, but the process can also result in alternative products (Figure 2) [4]. Breakdown mechanics are well summarized in [13]. Some ITCs, including 2-hydroxyisothiocyanates (2-OH-ITCs), are unstable and rearrange to oxazolidine-2-thiones spontaneously. Indole glucosinolates (IGSLs) also form unstable ITCs that are readily converted to various downstream products which are not dealt with here.

The scope of the current review are the effects of isothiocyanates on fungi. These natural products are the main antifungal products of a well-studied chemical defense system, which presents glucosinolates as core chemical structures. It is important to note that despite some have relevance in *Brassicaceae* as pathogens, data on oomycetes, rhizaria and other non-fungal microorganisms were not included in the current paper. Additional information about the search queries and bibliography processing are provided in Supplementary File S1 and Table S1.

## 2. Direct Antifungal Effects of Glucosinolate-Derived Isothiocyanates

The antifungal effects of ITC were described as early as 1966 [14]. Since then, a growing scientific literature shows an abundance of articles that deal with antifungal effects of various ITCs against different fungi. The compounds are typically added either as pure compounds or extracts of ITC-containing plants. In the latter case, ITCs are formed in situ by myrosinase enzymes, added in purified form in some studies or, alternatively, the plant’s own enzyme activity is relied upon to do the conversion. The effects of pure ITCs on fungi are summarized in Table 1. List of activities include inhibition of growth, germination of sclerotia or spores as well as biofilm formation. Activity against various plant pathogens, human pathogens, mycotoxigenic fungi and other (wood-decay fungi, insect pathogenic strains) was described. The most researched fungi are plant pathogens, including *Rhizoctonia solani*, *Sclerotinia sclerotiorum*, *Alternaria brassicicola*, *Fusarium oxysporum*, while human-pathogenic strains include species from the genus *Candida*. The most frequently tested ITC is allyl isothiocyanate, a common *Brassicaceae* compound. Other ITCs include some of natural origin, like benzyl isothiocyanate, sulphoraphane, 2-phenylethyl isothiocyanate (or phenethyl isothiocyanate), but some studies tested synthetic ITCs that do not occur naturally [15,16].

In several instances, the ITCs were administered as plant extracts or homogenizates. These studies are also suitable for detection of antifungal activity and can be the method of choice when a chemically non-characterized plant is examined for the first time, or, if the ITCs in the plant are not available from vendors. Nevertheless, this approach requires chemical characterization of the plant volatiles, and there is no means to subtract synergistic activity from the seen phenomenon. The list of ITC-containing, chemically characterized plant matrices used to test antifungal activity are summarized in Table 2. The same activities were tested as in the case of pure ITCs; in fact, several well-designed studies did both approaches [17,18]. As it can be seen from Table 2, this approach enables coverage of a much wider set of ITCs, at the cost of no data on the potency of individual components: various methylthioalkyl and methylsulfinylalkyl ITCs [19,20], a glycosylated ITC [21] were included as well. Tested organisms belonged to the same classes. Additional notable species include a much wider set of *Aspergillus* spp. and *Penicillium*, along with data on the human pathogenic *Trichophyton* spp. [21,22,23] and *Malassezia* spp. [24], and on the plant pathogen *Verticillium dahliae* [25,26]. The compound sources were mostly *Brassicaceae* plant tissues, usually *Brassica* spp. (typically one or more of *B. juncea*, *B. rapa*, *B. oleracea*, *B. nigra*, *B. carinata*) and *Sinapis* spp. (mostly, *S. alba*). A few studies carried out a chemical analysis of less widespread *Brassicaceae* plants, such as *Cardaria draba* [19], *Aurinia sinuata* [27], *Iberis amara* [28], or *Bunias orientalis* [20]. There are data on a few non-*Brassicaceae* plants as well, e.g., *Moringa oleifera* (Moringaceae) [21,29], and *Carica papaya* (Caricaceae) [30].

A handful of studies studied *Brassicaceae* plant extracts’ effects without chemical characterization of the extracts themselves. These are summarized in Table S2. The plants all belong to the *Brassicaceae* family; in a few instances, seed meals that also contains GSLs were tested. The tested phenomena include growth inhibition of various fungi (same functional groups as in Table 1 and Table 2), except the results of [31] who showed growth stimulation of *Brassica rapa* extracts on ectomycorrhizal fungi *Paxillus* spp. In this case it is unclear whether the extract was free of ITCs, or the antifungal activity was offset by other compounds in the extract.

Being biodegradable, highly active and of natural origin, ITCs are often considered attractive viable alternatives to other antifungal agents in various applications in the food industry and agriculture. These applications are detailed in Section 7.

**Table 1:** A review of direct effects of purified, standard and synthesized (but naturally occurring) isothiocyanates in in vitro models using in-medium or vapor exposure. Reoccurring genus names in the same cells are abbreviated.

Isothiocyanate	Source of ITC	Fungal Strains	Fungus Functions	Activity	Ref.
**Aliphatic**					
2(R)-2OH-3BuITC	*Brassica napus* GSL + *Sinapis alba* MYR	*Botrytis cinerea, Monilinia laxa, Mucor piriformis, Penicillium expansum*	PP	CG	[32]
2(S)-2OH-3BuITC	*Crambe abyssinica* GSL + *S. alba* MYR	*Alternaria alternata, Botrytis cinerea, Colletotricum coccodes, Diaporthe phaseolorum, Fusarium culmorum, F. oxysporum, Rhizoctonia solani, Sclerotinia sclerotiorum*	PP	MG	[6]
3-BuITC	standard	*Metarhizium anisopliae*	EP	CG, MG	[33]
3-BuITC	std.	*Bipolaris sorokiniana, Fusarium graminearum, Gaeumannomyces graminis, Rhizoctonia solani*	PP	MG	[34]
3-BuITC	*B. napus* GSL + *S. alba* MYR	*Monilinia laxa, Mucor piriformis*	PP	CG	[32]
3-BuITC	*Brassica rapa* GSL + *S. alba* MYR	*Fusarium culmorum*	PP	MG	[6]
3-BuITC	*Brassica* plant GSL + *S. alba* MYR	*Leptosphaeria maculans*	PP	MG	[35]
3-BuITC	std.	*Alternaria brassicae, Sclerotinia sclerotiorum*	PP	MG	[18]
4-ITCBuA	synthesized	*Candida albicans*	HR	MG	[36]
4-PeITC	std.	*Bipolaris sorokiniana, Fusarium graminearum, Gaeumannomyces graminis, Rhizoctonia solani*	PP	MG	[34]
4-PeITC	std.	*Aspergillus niger, Candida holmii, Saccharomyces cerevisiae*	ENV	MG	[37]
4-PeITC	std.	*Alternaria brassicae, Sclerotinia sclerotiorum*	PP	MG	[18]
AITC	std.	*Rhizoctonia solani*	PP	MG	[17]
AITC	std.	*Sclerotium rolfsii, Sclerotinia sclerotiorum*	PP	SCG	[38]
AITC	std.	*Aspergillus parasiticus, Penicillium expansum*	MT	MG	[39]
AITC	std.	*Aspergillus flavus, Botrytis cinerea, Penicillium expansum*	PP, MT	CG, MG	[40]
AITC	std.	*Alternaria brassicae, A. brassicicola*	PP	CG, MG	[41]
AITC	std.	*Fusarium oxysporum*	PP	CG, MG, SPG	[42]
AITC	std.	*Aspergillus niger*	PP	MG	[43]
AITC	std.	*Metarhizium anisopliae*	EP	CG, MG	[33]
AITC	std.	*Colletotricum coccodes, Helminthosporium solani, Rhizoctonia solani*	PP	MG	[44]
AITC	std.	*Alternaria alternata, Aspergillus parasiticus, Fusarium tricinctum, F. verticilloides, Gibberella zeae*	MT	mycotoxin production	[45]
AITC	std.	*Candida albicans*	HR	MG, biofilm formation	[46]
AITC	std.	*Sclerotinia sclerotiorum*	PP	MG	[47]
AITC	std.	*Pleiochaeta setosa*	PP	MG	[48]
AITC	std.	*Geotrichum citri-aurantii*	PP	CG, MG	[49]
AITC	std.	*Fusarium oxysporum, Macrophomina phaseolina, Oidiodendron cerealis, Paraphoma radicina, Setophoma terrestris*	endophyte	MG	[50]
AITC	std. & synt.	*Alternaria tenuis, Aspergillus flavus, A. fumigatus, A. niger, A. oryzae, Cephalothecium roseum, Cladosporium herbarum, Cytospora sp., Fusarium sp., Monilia sitophila, P. brevicompactum, P. cyclopium, Rhizopus oryzae, Schizophyllum commune, Trichoderma viride, Trichophyton gypseum*	PP, ENV, HR	MG	[51]
AITC	std.	*Bipolaris sorokiniana, Fusarium graminearum, Gaeumannomyces graminis, Rhizoctonia solani*	PP	MG	[34]
AITC	std.	*Candida albicans*	HR	MG	[52]
AITC	std.	*Aspergillus niger, Candida holmii, Saccharomyces cerevisiae*	ENV	MG	[37]
AITC	std.	*Botrytis cinerea, Penicillium expansum*	PP	CG, MG	[53]
AITC	std.	*Penicillium notatum*	ENV	MG	[54]
AITC	*Brassica juncea* GSL + *S. alba* MYR	*Botrytis cinerea, Monilinia laxa, Mucor piriformis, Penicillium expansum, Rhizopus stolonifer*	PP	CG	[32]
AITC	*B. juncea* GSL + *S. alba* MYR	*Alternaria alternata, Botrytis cinerea, Colletotricum coccodes, Diaporthe phaseolorum, Fusarium culmorum, F. oxysporum, Rhizoctonia solani, Sclerotinia sclerotiorum*	PP	MG	[6]
AITC	std. sinigrin + std. MYR	*Saccharomyces cerevisiae*	N.I.	MG	[55]
AITC	std.	*Candida albicans*	HR	MG	[56]
AITC	*B. juncea* GSL + *S. alba* MYR	*Rhizoctonia solani*	PP	soil colonisation	[28]
AITC	*Brassica* plant GSL + *S. alba* MYR	*Leptosphaeria maculans*	PP	MG	[35]
AITC	std.	*Gaeumannomyces graminis*	PP	MG	[57]
AITC	std.	*Glomus clarum*	ENV	SPG	[58]
AITC	std.	*Rhizoctonia solani*	PP	MG	[17]
AITC	std.	*Rhizoctonia solani*	PP	MG, SCG	[59]
AITC	std.	*Sclerotium rolfsii*	PP	MG	[60]
AITC	std.	*Sclerotium rolfsii*	PP	MG	[61]
AITC	std. sinigrin + std. MYR	*Verticillium longisporum*	PP	MG	[25]
AITC	std.	*Candida albicans*	HR	MG	[62]
AITC	std.	*Fusarium oxysporum, Rhizoctonia solani*	PP	MG	[63]
AITC	std.	*Beauveria bassiana, Isaria fumosorosea*	EP	CG	[64]
AITC	std.	*Aspergillus flavus, A. niger, A. ochraceus, Botryotinia fuckeliana, Fusarium oxysporum, Geotrichum spp., Penicillium expansum, P. roqueforti, P. verrucosum, Rhizopus stolonifer*	ENV	MG	[65]
AITC	std.	*Aspergillus fumigatus, A. nomius, A. niger, Candida albicans, Cryptococcus neoformans, Eupenicillum hirayamae, Penicillium cinna-mopurpureum, P. expansum, P. viridicatum, Trichophyton rubrum*	HR, ENV	MG	[66]
AITC	std.	*Aspergillus niger, A. ochraceus, Penicillium citrinum*	ENV	MG	[67]
AITC	std.	*Candida albicans*	ENV	MG	[68]
AITC	std.	*Botrytis cinerea*	PP	CG, MG	[69]
AITC	std.	*Aspergillus flavus*	MT	MG, mycotoxin production	[70]
AITC	std.	*Alternaria brassicae, Sclerotinia sclerotiorum*	PP	MG	[18]
AITC	std. sinigrin + transgenic MYR	*Rhizoctonia solani, Sclerotium rolfsii, Sclerotinia sclerotiorum*	PP	MG	[71]
AITC	std.	*Aspergillus niger, Aureobasidium pullulans, Fomitopsis palustris, Gliocladium virens, Penicillium funiculosurn, Rhizopus stolonifer, Trametes versicolor*	ENV	regrowth on wood specimens	[72]
AITC	std.	*Aspergillus niger*	PP	MG, SPG	[73]
AITC	std.	*Phymatotrichopsis omnivora*	PP	MG	[74]
AITC	std.	*Fusarium oxysporum, Pestalotiopsis spp., Rhizoctonia solani, Verticillium dahliae*	PP	MG	[75]
AITC	std.	*Aspergillus flavus*	ENV	MG	[76]
AITC	std.	*Penicillium nordicum*	ENV	MG	[77]
AITC	std.	*Aspergillus parasiticus*	MT	MG	[78]
AITC	std.	*Alternaria alternata*	PP	MG	[79]
BuITC	std.	*Fusarium oxysporum*	PP	CG, SPG	[42]
BuITC	std.	*Metarhizium anisopliae*	EP	CG, MG	[33]
BuITC	std.	*Sclerotinia sclerotiorum*	PP	MG, SCG	[47]
BuITC	std.	*Geotrichum citri-aurantii*	PP	CG, MG	[49]
BuITC	std.	*Alternaria tenuis, Aspergillus flavus, A. fumigatus, A. niger, A. oryzae, Cladosporium herbarum, Monilia sitophila, Penicillium brevicompactum, P. cyclopium, Trichoderma viride*	PP, ENV	MG	[51]
BuITC	synt.	*Rhizoctonia solani*	PP	MG	[80]
BuITC	std.	*Phymatotrichopsis omnivora*	PP	MG	[74]
EITC	std.	*Fusarium oxysporum*	PP	CG, MG, SPG	[42]
EITC	std.	*Sclerotinia sclerotiorum*	PP	MG, SCG	[47]
EITC	std.	*Geotrichum citri-aurantii*	PP	CG, MG	[49]
EITC	std. & synt.	*Aspergillus niger*	PP	MG	[51]
EITC	synt.	*Rhizoctonia solani*	PP	MG	[80]
EITC	std.	*Botrytis cinerea, Penicillium expansum*	PP	CG, MG	[53]
HexITC	std. & synt.	*Aspergillus niger, Penicillium cyclopium, Rhizopus oryzae*	PP	MG	[51]
HexITC	std.	*Candida albicans*	HR	MG	[52]
HexITC	synt.	*Rhizoctonia solani*	PP	MG	[80]
iBuITC	std. & synt.	*Aspergillus niger, Penicillium cyclopium, Rhizopus oryzae*	PP	MG	[51]
iPrITC	std.	*Colletotricum coccodes, Helminthosporium solani, Rhizoctonia solani*	PP	MG	[44]
iPrITC	std. & synt.	*Aspergillus niger, Penicillium cyclopium, Rhizopus oryzae*	PP	MG	[51]
MeITC	std.	*Colletotricum coccodes, Helminthosporium solani*	PP	MG	[44]
MeITC	std.	*Geotrichum citri-aurantii*	PP	CG, MG	[49]
MeITC	std. & synt.	*Alternaria tenuis, Aspergillus flavus, A. fumigatus, A. niger, A. oryzae, Cladosporium herbarum, Monilia sitophila, Penicillium brevicompactum, P. cyclopium, Trichoderma viride*	PP, ENV	MG	[51]
MeITC	std.	*Bipolaris sorokiniana, Fusarium graminearum, Gaeumannomyces graminis, Rhizoctonia solani*	PP	MG	[34]
MeITC	synt.	*Rhizoctonia solani*	PP	MG	[80]
MeITC	std.	*Gaeumannomyces graminis*	PP	MG	[57]
PeITC	std.	*Metarhizium anisopliae*	EP	CG, MG	[33]
PeITC	std. & synt.	*Aspergillus niger, Penicillium cyclopium, Rhizopus oryzae*	PP	MG	[51]
PeITC	synt.	*Rhizoctonia solani*	PP	MG	[80]
PrITC	std.	*Metarhizium anisopliae*	EP	CG, MG	[33]
PrITC	std.	*Colletotricum coccodes, Helminthosporium solani, Rhizoctonia solani*	PP	MG	[44]
PrITC	std. & synt.	*Aspergillus niger, Penicillium cyclopium, Rhizopus oryzae*	PP	MG	[51]
PrITC	synt.	*Rhizoctonia solani*	PP	MG	[80]
**Aromatic**					
2-OHPEITC	*Barbarea vulgaris* GSL + *S. alba* MYR	*Fusarium culmorum*	PP	MG	[6]
3-MeOBnITC	synt.	*Aspergillus fumigatus, Candida albicans*	HR, ENV	MG	[81]
3-MeOBnITC	std.	*Verticillium dahliae*	PP	MG	[82]
3-MeOBnITC	*Salvadora persica*	*Aspergillus niger, Candida albicans*	HR	MG	[83]
3-OHBnITC	*S. persica*	*Aspergillus niger*	HR	MG	[83]
3-PPrITC	synt.	*Aspergillus brasiliensis, Candida albicans*	HR, ENV	MG	[84]
4-Ac-α-L-RhaBnITC	*Moringa oleifera*	*Epidermophyton floccosum, Trichophyton rubrum*	HR	MG	[21]
4-MeOBnITC	std.	*Aspergillus fumigatus, Candida albicans, C. crusei, C. glabrata, C. parapsilosis, Cryptococcus neoformans*	HR	MG	[85]
4-MeOBnITC	synt.	*Aspergillus fumigatus, Candida albicans*	HR, ENV	MG	[81]
4-MeOBnITC	std. & synt.	*Alternaria tenuis, Aspergillus flavus, A. fumigatus, A. niger, A. oryzae, Cladosporium herbarum, Monilia sitophila, Penicillium brevicompactum, P. cyclopium, Trichoderma viride*	PP, ENV	MG	[51]
4-OHBnITC	*S. alba* GSL + *S. alba* MYR	*Botrytis cinerea, Monilinia laxa, Mucor piriformis, Penicillium expansum, Rhizopus stolonifer*	PP	CG	[32]
4-OHBnITC	*S. alba* GSL + *S. alba* MYR	*Botrytis cinerea, Monilinia laxa, Mucor piriformis, Penicillium expansum, Rhizopus stolonifer*	PP	curative activity	[86]
4-OHBnITC	*S. alba* GSL + *S. alba* MYR	*Fusarium culmorum*	PP	MG	[6]
4-OHPEITC	std.	*Aspergillus fumigatus, Candida albicans*	HR	MG	[85]
4-PBuITC	synt.	*Aspergillus brasiliensis, Candida albicans*	HR, ENV	MG	[84]
4-α-L-RhaBnITC	*Moringa oleifera*	*Epidermophyton floccosum, Trichophyton rubrum*	HR	MG	[21]
4-α-L-RhaBnITC	*Moringa oleifera*	*Aspergillus niger, Candida albicans*	HR, ENV	MG	[87]
5-PPeITC	synt.	*Aspergillus brasiliensis, Candida albicans*	HR, ENV	MG	[84]
BnITC	std.	*Alternaria brassicae, A. brassicicola*	PP	CG, MG	[41]
BnITC	std.	*Candida albicans, C. glabrata, C. krusei, C. parapsilosis, C. tropicalis*	HR	MG	[88]
BnITC	std.	*Alternaria alternata*	PP	MG, SPG, mycotoxin production	[89]
BnITC	std.	*Fusarium oxysporum*	PP	CG, SPG	[42]
BnITC	std.	*Colletotricum coccodes, Helminthosporium solani*	PP	MG	[44]
BnITC	synt.	*Aspergillus fumigatus, Candida albicans*	HR, ENV	MG	[81]
BnITC	std.	*Sclerotinia sclerotiorum*	PP	MG, SCG	[47]
BnITC	std.	*Geotrichum citri-aurantii*	PP	CG, MG	[49]
BnITC	std. & synt.	*Alternaria tenuis, Aspergillus flavus, A. niger, A. oryzae, Cephalothecium roseum, Cladosporium herbarum, Cytospora sp., Fusarium sp., Monilia sitophila, Penicillium brevicompactum, P. cyclopium, Rhizopus oryzae, Schizophyllum commune, Trichoderma viride, Trichophyton gypseum*	PP, ENV	MG	[51]
BnITC	std.	*Bipolaris sorokiniana, Fusarium graminearum, Gaeumannomyces graminis, Rhizoctonia solani*	PP	MG	[34]
BnITC	std.	*Candida albicans*	HR	MG	[52]
BnITC	std.	*Aspergillus niger, Candida holmii, Saccharomyces cerevisiae*	ENV	MG	[37]
BnITC	*Lepidium sativum* GSL + *S. alba* MYR	*Monilinia laxa, Mucor piriformis*	PP	CG	[32]
BnITC	*L. sativum* GSL + *S. alba* MYR	*Alternaria alternata, Botrytis cinerea, Colletotricum coccodes, Diaporthe phaseolorum, Fusarium culmorum, F. oxysporum, Rhizoctonia solani, Sclerotinia sclerotiorum*	PP	MG	[6]
BnITC	std.	*Aspergillus spp., Candida spp., Cryptococcus neoformans, Fonsecaea pedrosoi, Fusarium solani, Microsporum canis, Pseudallescheria boydii, Saccharomyces cerevisiae, Sporothrix schenckii, Trichophyton rubrum*	HR, ENV	MG	[23]
BnITC	synt.	*Aspergillus brasiliensis, Candida albicans*	HR, ENV	MG	[84]
BnITC	std.	*Alternaria brassicae, Sclerotinia sclerotiorum*	PP	MG	[18]
BnITC	*Salvadora persica*	*Aspergillus niger*	HR	MG	[83]
BnITC	std.	*Phymatotrichopsis omnivora*	PP	MG	[74]
BnITC	std.	*Aspergillus parasiticus*	MT	MG	[78]
BnITC	std.	*Alternaria alternata*	PP	MG	[79,90]
PEITC	std.	*Fusarium oxysporum*	PP	CG, SPG	[42]
PEITC	std.	*Metarhizium anisopliae, Tolypocladium cylindrosporum*	EP	MG	[91]
PEITC	std.	*Alternaria brassicae, A. macrospora, Aspergillus niger, Bipolaris sorokiniana, Fusarium spp., Gaeumannomyces graminis, Lasiodiplodia theobromae, Pleiochaeta setosa, Rhizoctonia solani, Sclerotinia spp., Sclerotium rolfsii, Thielaviopsis basicola, Trichoderma sp.*	PP, ENV	MG	[92]
PEITC	std.	*Metarhizium anisopliae*	EP	CG, MG	[33]
PEITC	std.	*Colletotricum coccodes, Helminthosporium solani, Rhizoctonia solani*	PP	MG	[44]
PEITC	std.	*Sclerotinia sclerotiorum*	PP	MG, SCG	[47]
PEITC	std.	*Geotrichum citri-aurantii*	PP	CG, MG	[49]
PEITC	std.	*Fusarium oxysporum, Macrophomina phaseolina, Paraphoma radicina, Setophoma terrestris, Oidiodendron cerealis*	endophyte	MG	[50]
PEITC	std. & synt.	*Aspergillus niger, Penicillium cyclopium, Rhizopus oryzae*	PP	MG	[51]
PEITC	std.	*Bipolaris sorokiniana, Fusarium graminearum, Gaeumannomyces graminis, Rhizoctonia solani*	PP	MG	[34]
PEITC	std.	*Candida albicans*	HR	MG	[52]
PEITC	std.	*Aspergillus niger, Candida holmii, Saccharomyces cerevisiae*	ENV	MG	[37]
PEITC	std.	*Aspergillus niger, Candida albicans, Penicillium citrinum*	ENV	MG	[93]
PEITC	std.	*Candida albicans*	HR	MG	[56]
PEITC	std.	*Gaeumannomyces graminis*	PP	MG	[57]
PEITC	std.	*Rhizoctonia solani*	PP	MG, SCG	[59]
PEITC	synt.	*Aspergillus brasiliensis, Candida albicans*	HR, ENV	MG	[84]
PEITC	std.	*Alternaria brassicae, Sclerotinia sclerotiorum*	PP	MG	[18]
PEITC	std.	*Alternaria alternata*	PP	MG	[79]
PEITC	std.	*Alternaria alternata*	PP	MG, SPG	[94]
PITC	std.	*Fusarium oxysporum*	PP	CG, SPG	[42]
PITC	std.	*Phymatotrichopsis omnivora*	PP	MG	[74]
PITC	std.	*Sclerotinia sclerotiorum*	PP	MG, SCG	[47]
PITC	std.	*Candida albicans*	HR	MG	[52]
PITC	std.	*Saccharomyces cerevisiae*	N.I.	MG	[55]
PITC	std.	*Aspergillus parasiticus*	MT	MG	[78]
PITC	std.	*Alternaria alternata*	PP	MG	[79]
**Indole**					
1-MeO-3-IMeITC	*Brassica plant* GSL + *S. alba* MYR	*Leptosphaeria maculans*	PP	MG	[35]
3-IMeITC	*Brassica plant* GSL + *S. alba* MYR	*Leptosphaeria maculans*	PP	MG	[35]
rapalexin A	synt.	*Alternaria brassicicola*	PP	MG	[95]
sinapigladioside	insect symbiont *Burkholderia gladioli*	*Purpureocillium lilacinum*	EP	MG	[96]
**Sulfur containing**					
3-MeSOOPrITC	std. & synt.	*Aspergillus niger, Penicillium cyclopium, Rhizopus oryzae*	PP	MG	[51]
3-MeSOOPrITC	std.	*Aspergillus niger, Candida holmii, Saccharomyces cerevisiae*	ENV	MG	[37]
3-MeSOOPrITC	*Cheirantus annuus* GSL + *S. alba* MYR	*Fusarium culmorum*	PP	MG	[6]
3-MeSOPrITC	std.	*Candida albicans*	HR	MG	[52]
3-MeSOPrITC	std.	*Aspergillus niger, Candida holmii, Saccharomyces cerevisiae*	ENV	MG	[37]
3-MeSOPrITC	*Iberis amara* GSL + *S. alba* MYR	*Alternaria alternata, Botrytis cinerea, Colletotricum coccodes, Diaporthe phaseolorum, Fusarium culmorum, F. oxysporum, Rhizoctonia solani, Sclerotinia sclerotiorum*	PP	MG	[6]
3-MeSOPrITC	*I. amara* GSL + *S. alba* MYR	*Rhizoctonia solani*	PP	soil colonisation	[28]
3-MeSPrITC	std.	*Aspergillus niger, Candida holmii, Saccharomyces cerevisiae*	ENV	MG	[37]
4-MeSBuITC	std.	*Candida albicans*	HR	MG	[52]
4-MeSBuITC	*Eruca sativa* GSL + *S. alba* MYR	*Fusarium culmorum*	PP	MG	[6]
4-MeSBuITC	*E. sativa* GSL + *S. alba* MYR	*Rhizoctonia solani*	PP	soil colonisation	[28]
4-MeSO-3-BuITC	std.	*Candida albicans*	HR	MG	[52]
4-MeSO-3-BuITC	Raphanus sativus GSL + *S. alba* MYR	*Botrytis cinerea, Monilinia laxa, Mucor piriformis, Penicillium expansum, Rhizopus stolonifer*	PP	CG	[32]
4-MeSO-3-BuITC	*R. sativus* GSL + *S. alba* MYR	*Botrytis cinerea, Monilinia laxa, Mucor piriformis, Penicillium expansum, Rhizopus stolonifer*	PP	curative activity	[86]
4-MeSO-3-BuITC	*R. sativus* GSL + *S. alba* MYR	*Fusarium culmorum*	PP	MG	[6]
4-MeSOBuITC	synt.	*Cryptococcus neoformans*	HR	MG	[97]
4-MeSOBuITC	std.	*Nosema ceranae*	EP	SPG	[98]
4-MeSOBuITC	std.	*Candida albicans*	HR	MG	[52]
4-MeSOBuITC	std.	*Alternaria brassicae, Sclerotinia sclerotiorum*	PP	MG	[18]
4-MeSOOBuITC	std. & synt.	*Aspergillus niger, Penicillium cyclopium, Rhizopus oryzae*	PP	MG	[51]
5-MeSPeITC	std. & synt.	*Aspergillus niger, Penicillium cyclopium, Rhizopus oryzae*	PP	MG	[51]
9-MeSNonITC	std.	*Aspergillus niger, Candida holmii, Saccharomyces cerevisiae*	ENV	MG	[37]
9-MeSONonITC	std.	*Aspergillus niger, Candida holmii, Saccharomyces cerevisiae*	ENV	MG	[37]
9-MeSOONonITC	std.	*Aspergillus niger, Candida holmii, Saccharomyces cerevisiae*	ENV	MG	[37]
**Other**					
AITC, BnITC, PEITC mixture	std.	*Candida* spp.	HR	MG	[99]

Abbreviations: CG—conidia germination; ENV—environmental (decaying fungi, molds, etc.); HR—human related (pathogens, clinical isolates, dermatophytes, etc.); MG—mycelial growth; MT—mycotoxigenic; PF—perithecia formation; PP—plant pathogen; SCG—sclerotia germination; SPG—spore germination. Isothiocyanates: 1-MeO-3-IMeITC—1-methoxyindol-3-ylmethyl isothiocyanate; 2-OHPEITC—2-hydroxyphenethyl isothiocyanate; 2(R)-2OH-3BuITC—2(R)-2-hydroxy-3-butenyl isothiocyanate; 2(S)-2OH-3BuITC—2(S)-2-hydroxy-3-butenyl isothiocyanate; 3-BuITC—3-butenyl isothiocyanate; 3-IMeITC—indol-3-ylmethyl isothiocyanate; 3-MeOBnITC—3-methoxybenzyl isothiocyanate; 3-MeSOPrITC—3-(methylsulfinyl)propyl isothiocyanate; 3-MeSOOPrITC—3-(methylsulfonyl)propyl isothiocyanate; 3-MeSPrITC—3-(methylthio)propyl isothiocyanate; 3-OHBnITC—3-hydroxybenzyl isothiocyanate; 3-PPrITC—3-phenylpropyl isothioyanate; 4-α-L-RhaBnITC—4-(α-L-rhamnosyloxy)-benzyl isothiocyanate; 4-Ac-α-L-RhaBnITC—4-(4′-O-acetyl-α-L-rhamnosyloxy)-benzyl isothiocyanate; 4-ITCBuA—4-isothiocyanatobutanoic acid; 4-MeOBnITC—4-methoxybenzyl isothiocyanate; 4-MeSBuITC—4-(methylthio)butyl isothiocyanate (erucin); 4-MeSO-3-BuITC—4-methylsulfinyl-3-butenyl isothiocyanate (sulforaphene); 4-MeSOBuITC—4-(methylsulfinyl)butyl isothiocyanate (sulforaphane); 4-MeSOOBuITC—4-(methylsulfonyl)butyl isothiocyanate; 4-OHBnITC—4-hydroxybenzyl isothiocyanate; 4-OHPEITC—4-hydroxyphenethyl isothiocyanate; 4-PBuITC—4-phenylbutyl isothiocyanate; 4-PeITC—4-pentenyl isothiocyanate; 5-MeSPeITC—5-(methylthio)pentyl isothiocyanate (berteroin); 5-PPeITC—5-phenylpentyl isothiocyanate; 9-MeSNonITC—9-(methylthio)nonyl isothiocyanate; 9-MeSONonITC—9-(methylsulfinyl)nonyl isothiocyanate; 9-MeSOONonITC—9-(methylsulfonyl)nonyl isothiocyanate; AITC—allyl isothiocyanate; BnITC—benzyl isothiocyanate; BuITC—butyl isothiocyanate; EITC—ethyl isothiocyanate; HexITC—hexyl isothiocyanate; iBuITC—isobutyl isothiocyanate; iPrITC—isopropyl isothiocyanate; MeITC—methyl isothiocyanate; PeITC—pentyl isothiocyanate; PEITC—phenethyl isothiocyanate; PrITC—propyl isothiocyanate.

**Table 2:** A review of direct effects of isothiocyanate containing plant extracts in in vitro models using in-medium or vapor exposure. The concentration of isothiocyanates or glucosinolates were indicated in the manuscripts. Reoccurring genus names in the same cells are abbreviated.

Source	Major ITCs Detected	Fungal Strains	Fungus Functions	Activity	Ref.
*Apium graveolens*	iPrITC	*Fusarium oxysporum*	PP	MG	[100]
*Arabidopsis thaliana*	4-MePeITC	*Lecanicillium lecanii*	EP	CG	[101]
*Arabidopsis thaliana*	3-BuITC, 3-OHPrITC, 4-MeSOBuITC, AITC	*Verticillium longisporum*	PP	MG	[25]
*Armoracia rusticana*	3-BuITC, AITC, PEITC	*Epidermophyton floccosum, Microsporum canis, Trichophyton mentagrophytes, T. rubrum*	HR	MG	[22]
*Armoracia rusticana*	AITC, PEITC	*Ascosphaera apis*	EP	MG	[102]
*Armoracia rusticana*	AITC, PEITC, sBuITC	*Aspergillus niger, Candida albicans, Penicillium citrinum*	ENV	MG	[93]
*Armoracia rusticana*	AITC, PEITC	*Aspergillus fumigatus, A. nidulans, Candida albicans, Saccharomyces cerevisiae*	ENV, HR	MG	[56]
*Armoracia rusticana*	4-PeITC, 5-MeSOPeITC, 5-MeSPeITC, AITC, PEITC	*Aspergillus brasiliensis, Candida albicans*	ENV, HR	MG	[84]
*Armoracia rusticana*	3-BuITC, AITC, PEITC	*Candida albicans*	HR	MG	[62]
*Aurinia leucadea*	3-BuITC, 4-PeITC, 5-MeSOPeITC, sBuITC	*Candida albicans, Penicillium sp, Rhizopus stolonifer*	ENV, HR	MG	[103]
*Aurinia sinuata*	4-PeITC, 5-MeSOPeITC, 5-MeSPeITC	*Aspergillus niger, Candida albicans, Penicillium sp.*	ENV, HR, PP	MG	[27]
*Brassica campestris*	4-PeITC	*Rhizoctonia solani*	PP	MG	[17]
*Brassica carinata*	AITC	*Botrytis cinerea*	PP	CG, MG	[69]
*Brassica carinata*	AITC	*Fusarium sambucinum*	PP	MG	[26]
*Brassica juncea*	3-BuITC, AITC	*Rhizoctonia solani*	PP	MG	[104]
*Brassica juncea*	3-BuITC, AITC, sBuITC	*Rhizoctonia solani*	PP	MG	[17]
*Brassica juncea*	AITC	*Aspergillus parasiticus*	MT	MG	[105]
*Brassica juncea*	AITC	*Fusarium graminearum*	PP	MG	[106]
*Brassica juncea*	AITC	*Verticillium dahliae*	PP	MG	[107]
*Brassica juncea*	3-BuITC,3-MeSOPrITC,4-MeSBuITC,4-MeSOBuITC,5-MeSOPeITC, AITC	*Sclerotinia sclerotiorum*	PP	MG	[108]
*Brassica juncea*	AITC	*Bipolaris sorokiniana, Fusarium graminearum, Gaeumannomyces graminis, Rhizoctonia solani*	PP	MG	[109]
*Brassica juncea*	AITC, BnITC, PEITC	*Fusarium oxysporum, Sclerotinia sclerotiorum, Sclerotium cepivorum*	PP	SPG, SCG	[110]
*Brassica juncea*	AITC	*Sclerotium rolfsii*	PP	MG	[60]
*Brassica juncea*	AITC	*Sclerotinia sclerotiorum*	PP	MG	[111]
*Brassica juncea*	AITC	*Sclerotinia sclerotiorum*	PP	SCG	[112]
*Brassica juncea*	AITC	*Colletotrichum coccodes, Fusarium sambucinum, Rhizoctonia solani, Verticillium albo-atrum, V. dahliae*	PP	MG	[26]
*Brassica juncea*	AITC	*Fusarium graminearum, Fusarium poae*	PP	MG	[113]
*Brassica juncea*	PEITC	*Gaeumannomyces graminis*	PP	MG	[57]
*Brassica juncea and Sinapis alba mixture*	AITC	*Hypocrea lixii, Ilyonectria destructans, Mortierella alpina, Rhizoctonia solani*	PP	MG	[114]
*Brassica napus*	MeITC	*Gaeumannomyces graminis*	PP	MG	[57]
*Brassica napus*	PEITC	*Bipolaris sorokiniana, Fusarium graminearum, Gaeumannomyces graminis, Rhizoctonia solani*	PP	MG	[109]
*Brassica napus*	AITC, BnITC, PEITC	*Fusarium oxysporum, Sclerotium cepivorum*	PP	SPG, SCG	[110]
*Brassica napus* and *Brassica rapa* mixture	3-BuITC, 4-PeITC, 5-MeSPeITC, PEITC	*Rhizoctonia fragariae*	PP	MG	[115]
*Brassica nigra*	AITC	*Fusarium sambucinum*	PP	MG	[26]
*Brassica oleracea*	AITC	*Rhizoctonia solani*	PP	MG	[17]
*Brassica oleracea*	3-BuITC, AITC	*Rhizoctonia solani*	PP	MG	[104]
*Brassica oleracea*	2(R)-2OH-3BuITC,4-MeSBuITC,4-MeSOBuITC, AITC	*Candida albicans*	HR	MG	[116]
*Bunias orientalis*	4-MeSO-3-BuITC,4-OHBnITC, BuITC, iPrITC	*Alternaria brassicae, Botrytis cinerea*	PP	MG	[20]
*Cardaria draba*	4-MeSBuITC, 4-MeSOBuITC, 4-MeSOOBuITC	*Candida albicans, Penicillium sp., Rhizopus stolonifer,*	ENV, HR	MG	[19]
*Carica papaya*	BnITC	*Aspergillus amestelodanii, A. fumigatus, A. niger, Candida albicans, C. lipolytica, Cladosporium cladosporioides, Endomycopsis fibuliger, Gliocladium roseum, Mucor sp., Penicillium chrysogenum, P. cyclopium, P. digitatum, P. expansum, P. lilacinum, P. notatum, P. spinulosum, Saccharomyces cerevisiae, S. fragilis*	ENV, HR, PP	MG	[30]
*Degenia velebitica*	4-PeITC	*Candida albicans*	HR	MG	[117]
*Diplotaxis harra*	3-BuITC, iPrITC	*Aspergillus niger, Fusarium oxysporum, Kluyveromyces lactis, Saccharomyces cerevisiae*	ENV, PP	MG	[118]
*Eruca sativa*	4-MeSBuITC	*Sclerotinia sclerotiorum*	PP	SCG	[112]
*Eruca sativa*	4-MeSBuITC	*Malassezia furfur, Microsporum canis, Trichophyton mentagrophytes*	HR	MG	[24]
*Erucaria microcarpa*	BnITC, BuITC, iPrITC	*Aspergillus niger, Fusarium oxysporum, Kluyveromyces lactis, Saccharomyces cerevisiae*	ENV, PP	MG	[118]
*Erysimum corinthium*	3-MeCOPrITC, 3-MeSOOPrITC, 3-MeSOPrITC, AITC	*Candida albicans*	HR	MG	[119]
*Lepidium latifolium*	AITC, sBuITC	*Candida albicans*	ENV	MG	[68]
*Moringa oleifera*	4-α-L-RhaBnITC	*Aspergillus oryzae, Botrytis allii, Candida pseudotropicalis, C. reukaufii, Coniophora cerebella, Fusarium oxysporum, Penicillium expansum, Piricularia oryzae, Polystictus versicolor, Saccharomyces carlsbergensis, Zygorrhynchus sp.*	ENV, HR, PP	MG	[29]
*Raphanus sativus*	4-MeS-3-BuITC, 4-MeSO-3-BuITC	*Candida albicans*	HR	MG	[52]
*Raphanus sativus*	4-MeSO-3-BuITC	*Sclerotinia sclerotiorum*	PP	SCG	[112]
*Raphanus sativus*	3-MeSO-3-BuITC, 2-OH-4-PeITC	*Candida albicans*	HR	MG	[116]
*Salvadora persica*	BnITC, 3-MeOBnITC, 3-OHBnITC	*Aspergillus niger, Candida albicans*	HR	MG	[83]
*Sinapis alba*	4-OHBnITC	*Fusarium graminearum*	PP	MG	[106]
*Sinapis alba*	4-OHBnITC	*Fusarium graminearum*	PP	CG, MG, PF, SG	[120]
*Sinapis alba*	4-OHBnITC	*Sclerotinia sclerotiorum*	PP	SCG	[112]
*Sinapis alba*	AITC	*Candida albicans*	HR	MG	[116]
*Sinapis alba*	AITC, BnITC, PEITC	*Fusarium oxysporum, Sclerotium cepivorum*	PP	SPG, SCG	[110]
*Sisymbrium officinale*	iPrITC, sBuITC	*Aspergillus niger, Candida albicans, Penicillium sp., Saccharomyces cerevisiae,*	ENV, HR, PP	MG	[121]
*Tropaeolum pentaphyllum*	BnITC	*Aspergillus flavus, A. fumigatus, A. niger, Candida albicans, C. dubliniensis, C. glabrata, C. guilliermondii, C. parapsilosis, C. tropicalis, Cryptococcus neoformans, Fonsecaea pedrosoi, Fusarium solani, Microsporum canis, Pseudallescheria boydii, Saccharomyces cerevisiae, Sporothrix schenckii, Trichophyton rubrum*	HR, ENV	MG	[23]
*Wasabia japonica*	AITC	*Beauveria bassiana, Isaria fumosorosea*	EP	CG	[64]

Abbreviations: CG—conidia germination; ENV—environmental (decaying fungi, molds, etc.); HR—human related (pathogens, clinical isolates, dermatophytes, etc.); MG—mycelial growth; PF—perithecia formation; PP—plant pathogen; SCG—sclerotia germination; SPG—spore germination. Isothiocyanates: 2(R)-2OH-3BuITC—2(R)-2-hydroxy-3-butenyl isothiocyanate; 3-BuITC—3-butenyl isothiocyanate; 3-MeCOPrITC—3-(methylcarbonyl)propyl isothiocyanate; 3-MeSOPrITC—3-(methylsulfinyl)propyl isothiocyanate; 3-OHPrITC—3-hydroxypropyl isothiocyanate; 4-α-L-RhaBnITC—4-(α-L-rhamnosyloxy)-benzyl isothiocyanate; 4-MePeITC—4-methylpentyl isothiocyanate; 4-MeS-3-BuITC—4-methylthio-3-butenyl isothiocyanate; 4-MeSBuITC—4-(methylthio)butyl isothiocyanate (erucin); 4-MeSO-3-BuITC—4-methylsulfinyl-3-butenyl isothiocyanate (sulforaphene); 4-MeSOBuITC—4-(methylsulfinyl)butyl isothiocyanate (sulforaphane); 4-OHBnITC—4-hydroxybenzyl isothiocyanate; 4-PeITC—4-pentenyl isothiocyanate; 5-MeSOPeITC—5-(methylsulfinyl)pentyl isothiocyanate; 5-MeSPeITC—5-(methylthio)pentyl isothiocyanate (berteroin); AITC—allyl isothiocyanate; BnITC—benzyl isothiocyanate; iPrITC—isopropyl isothiocyanate; MeITC—methyl isothiocyanate; PEITC—phenethyl isothiocyanate; sBuITC—sec-butyl isothiocyanate.

## 3. Proposed Mechanisms of the Isothiocyanate Antifungal Activity

### 3.1. The Role of Isothiocyanate Reactivity in Bioactivity

**Figure 3:**
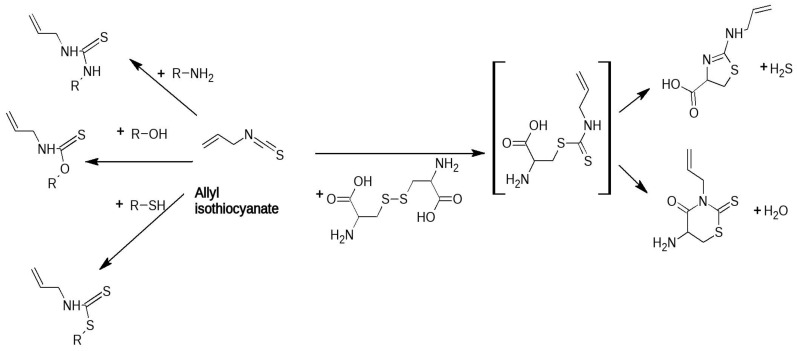
Reaction of isothiocyanates with nucleophiles. Compiled from data in [13].

ITCs most likely act through their chemical reactivity. They contain a very electrophilic carbon atom that is reactive towards thiols, amines and alcohols to yield dithiocarbamates, thiourea or *O*-thiocarbamate derivatives, respectively (Figure 3) [13]. Proteins, peptides and amino acids therefore contain sites suitable for an attack by ITCs, resulting in thioureas at the amino group [122]. A relatively early finding by [123] already supported the idea that reactivity is required for antifungal activity. The authors showed that the germination of the vesicular-arbuscular mycorrhiza *Glomus intraradices* can be inhibited by ITCs, but this effect can be antagonized by adding compounds that are known to react with ITCs, such as glutathione (GSH), lysine or arginine. The reactivity also explains the findings of [37] who conducted a study on ITCs and found that the antifungal activity of ITCs was better than in nucleophile-poor media—in other words, media that consume the agent to a lesser extent. The effects were more prominent for the long-chain 9-(methylsulfinyl)nonyl ITC and 9-(methylsulfonyl)nonyl ITC. The activity difference was as much as 4-16-fold.

### 3.2. Possible Targets of ITCs

The reaction rate is 3-4 orders of magnitude faster with thiols compared to amines and alcohols [124]. Hence, two of most likely targets of ITCs are the abundant glutathione pool [125] and the thiol side chains of proteins, or in other words, the redox homeostasis. ITCs indeed induce oxidative stress in *C. albicans* as shown by [56], resulting in elevated superoxide content and upregulation of glutathione reductase, glutathione peroxidase, catalase and superoxide dismutase activities. Higher doses of ITCs completely depleted the GSH pool and killed the fungi. A synergism between 1-chloro-2,4-dinitrobenzene (a GSH depleting agent) and ITCs was also shown. Another study [126] on the toxicity of various (benzyl, phenethyl, allyl) ITCs in the Brassicaceae-pathogen *Alternaria brassicicola* found a decreased oxygen consumption rate, an increased intracellular accumulation of ROS and depolarization of the mitochondrial membrane, supporting the above hypotheses.

The other changes described can either be downstream effects of the above oxidative stress, or dysfunction of proteins that do not tolerate chemical changes on thiol side-chains. Studying a yeast knockout model, [127] has found that impaired vacuole function leads to increased vulnerability to sulforaphane. The compound was also shown to increase vacuolar pH, and higher vacuolar pH resulted in higher tolerance towards the compound [127]. The less polar phenethyl ITC was shown to cause a cell membrane disruption in *Alternaria alternata* [94]. The same was observed in case of *Alternaria alternata* treated with benzyl ITC [89]. More in-depth effects are detailed below.

### 3.3. Transcriptome-Level in Isothiocyanate-Exposed Fungi Reveal Defense Mechanisms

To study mechanisms on how fungi cope with oxidative stress and other dysfunctions caused by ITCs, the transcriptional response of *Alternaria brassicicola* (a specialist *Brassica* pathogen) to allyl ITC was examined in a study by [128]. About 35% of the differentially expressed (induced) annotated genes were stress and defense genes against oxidative stress such as glutathione S-transferases, γ-glutamylcysteine synthetases, thioredoxins, oxidoreducatases, heat-shock proteins, etc. A wide range of membrane transporters (PDR ABC transporter, MFS transporter analogues) accounted for another 16% of over-expressed genes. An increase in reactive oxygen species (ROS) was also observed. These data support the hypothesis that ITCs primarily act as oxidative stress agents by causing a redox imbalance via interference with GSH homeostasis. Interestingly, a positive-acting sulfur regulatory protein was also over-expressed, which suggest that the fungus might also use ITC as a source of sulfur. This might be the background of the interesting phenomenon that low amounts of allyl ITC increased the growth of the fungus in vitro.

The study of [126] used the same fungal species to study ITC effects on the transcriptome. The oxidative stress regulators MAP kinase AbHog1 and the transcription factor AbAP1 (a *Saccharomyces cerevisiae* YAP1 protein ortholog) were significantly upregulated during exposure, resulting in activation of further antioxidant genes: 10-100-fold induction in thioreductases, a quinone oxidoreductase, a glutathion peroxidase, glutathione transferases and a gamma-glutamylcysteine synthetase was observed in wild-type *A. brassicicola*. AbHog1 or AbAP1 mutants were shown to be hypersensitive to ITCs—these are also significantly less virulent pathogens in *Brassica* at the same time. In contrast, the wild-type fungus was able to cope with relatively high amounts of allyl ITC after a lag period: after a delay, almost normal growth speed was achieved, again suggesting that a key mechanism to cope with ITCs is the oxidative stress response machinery.

In a *Fusarium solani* model [129], allyl ITC also causes hyphal deformity and electrolyte leakage. A yeast-like vacuolar transient receptor potential channel regulator (FsYvc1, a STRPC family member) was shown to be involved in this mechanism behind sensitivity: loss of FsYvc1 results in hypersensitivity towards allyl ITC, accompanied by a 1.3–1.5-fold hyperaccumulation of ROS, but no changes in a variety of other tested parameters. The mutants also showed reduced glutathione-S-transferase expression when not exposed to allyl ITC compared with the wild-type, explaining the increased susceptibility.

Another paper [130] used *Sclerotinia sclerotiorum*, the stem rot pathogen of *Brassica napus* (oilseed rape) as the ITC exposure model organism, to study the gene expression response. Importantly, fungi repeatedly exposed to either hydrated mustard powder (containing both GSL and myrosinase) or synthetic ITCs develop tolerance towards ITC. The strain’s ability to grow was restored from complete inhibition to 80–85% growth speed at the highest allyl ITC dosage, accompanied by induction of glutathione S-transferase-like genes, and a 2-fold increase in glutathione S-transferase catalytic activity, suggesting a specific detoxification mechanism. An interesting study of the transcriptome of an allyl ITC-resistant *Alternaria alternata* strain by [131] has concluded that allyl ITC can induce tolerance/resistance mechanisms in the same manner as synthetic fungicides. Induced genes coding similar-to-known proteins included many proteins and enzymes involved in the activation of signal cascades promoting cell repair and maintenance, the overexpression of which result in generic resistance-like traits. Example groups include genes of biosynthesis of proteins and ribosomal subunits, amino acid biosynthesis, genome and nuclear structure organization, DNA damage response activities, chaperons. Importantly, some are similar to genes induced in fungi after azole fungicide exposure.

Some results point towards a more generic stress response issue: the upregulation of *msnA* (a putative stress regulatory gene) and the downregulation of *ap-1* (a bZip transcription factor involved in oxidative stress response) and *cat-2* (catalase, an antioxidant enzyme) was detected upon exposure to allyl ITC in *Aspergillus flavus* [70]. The loss of *msnA* resulted in increased production of conidia, aflatoxins and kojic acid in *Aspergillus*, suggesting that allyl ITC causes a misregulation of genes involved in the oxidative stress response.

### 3.4. Metabolism and Detoxification of Isothiocyanates by Fungi

ITCs are efficient antifungal agents, but despite this fact, several fungi have found ways to detoxify surprisingly large amounts. What is more, specific detoxification mechanisms were also described in fungi that live in close contact with Brassicaceae.

Aspecific modifications on the thiomethyl alkyl side-chains include S-oxidation of e.g., 4-(methylthio)butyl ITC to sulforaphane by the ascomycota fungus *Helminthosporium species* [132]. The fungus *Mortierella isabellina* can also do this biotransformation, but while the former metabolized the compounds into predominantly (S) isomers of sulfoxides, *M. isabellina* generated (R) isomers [133]. Note that this modification does not modify the moiety responsible for antifungal activity.

What are of more interest are the possible detoxification processes, which begin with a generic step in *Colletotrichum dematium* and *C. higginsianum* [134]: rapalexin A was metabolized into Cys conjugates. Both fungi metabolized the conjugate further to either a cyclic dithiocarbamate or an S-containing heterocyclic ring system, but the two fungal species use different metabolism pathways as the main transformation route. Compared to rapalexin A, the latter compound was shown to be a much weaker antifungal agent against both fungi and against a non-Brassicaceae strain of *Colletotrichum lentis*.

Several fungal genera were proven to be more successful than others in the capacity to deal with ITCs. This is highlighted by the work of [135] who tested various *Brassica* extracts and ITCs for fungitoxic activity against fungal isolates from *Brassiceaeae* plants and isolates from non-Brassicaceae plants in vitro. Higher tolerance towards ITCs was found in *Rhizopus* and *Fusarium*, the dominant fungal genera of the *Brassicaceae* rhizoplane, compared to members of the same genera from other plants. In the work of [50], endophytes from *Armoracia rusticana* (*Brassicaceae*) were shown to be more tolerant towards allyl ITC than soil fungi from the same soil.

Adapted strains might show specific detoxification mechanisms as well, as presented in the recent study of [136]. The necrotrophic mold *Sclerotinia sclerotiorum* was shown to detoxify ITCs by two independent pathways: conjugation to glutathione, followed by conversion into an N-Ac-Cys derivate; and hydrolysis to amines, followed by biotransformation into acetamides. The latter pathway was found to be dominant versus the former at a ratio of about 100:1. The enzyme responsible for the conversion was successfully produced in *E. coli* and shown to accept all tested ITCs (4-methylsulfoxybutyl ITC, 8-(methylsulfonyl)octyl ITC, and phenethyl ITC) as substrates, converting them into amines. Contrary to the ITCs, the corresponding amines are not inhibitory to the growth of the fungus. As the deletion of the gene reduces tolerance towards ITCs as well as fungal pathogenicity, it was concluded that the gene is in part responsible for the virulence of *S. sclerotiorum*.

A widely used, generic solution is the usage of various fungal glutathione S-transferases (GSTs) that likely play an important role in detoxification of ITCs if no other pathway is available. This biochemical route seems to be typical in the metabolic toolkit of fungi that live in close contact with plants that biosynthesize ITCs.

An interesting study [137] conducted genome-mining of *Alternaria brassicicola* and found 23 glutathione transferase sequences, a number comparable to that found in other necrotrophs (e.g., *Botrytis cinerea*) or hemibiotrophs (e.g., *Leptosphaeria maculans*). Only 17 of the 23 genes could be clustered into one of the previously defined fungal GST classes, the rest were ‘orphans’. The authors expressed some of the enzymes in *E. coli* that enabled a more detailed characterization, and quantified gene expression during in planta colonization. The enzymes fell into various categories along the following binary variables: (1) whether gene expression is induced during in planta pathogenesis; (2) whether being required for pathogenesis in planta; (3) whether its inactivation results in ITC hypersensitivity; (4) whether it accepts allyl ITC as a substrate. Several combinations of the above were observed for various genes/enzymes. The variety of available enzymes suggests the evolutionary approach of preparing with a wide arsenal of potential detoxificants, some of which are required to invade a plant with a specific chemical defense system. This is a logical consequence of the developments on the plant side—biosynthesizing a mixture of different natural products for antifungal defense.

Other fungi were described to contain glutathione S-transferases (GSTs): a fungal-specific one was isolated from *Phanerochaete chrysosporium* and characterized in [138]. A glutathione transferase of *Alternaria brassicicola* is significantly overexpressed when the fungus is exposed to ITCs, shown by [139]. The enzyme shows high transferase activity with allyl ITC and benzyl ITC. The enzyme is also upregulated during plant infection suggesting detoxification of ITCs. The *Trametes versicolor* glutathione transferases of class Omega 3S are also capable of catalysing conjugation of ITCs with GSH [140,141].

### 3.5. Efflux of Isothiocyanates

The fungus *Alternaria alternata* exposed to allyl ITC showed increased expression of amino acid permeases, the ABC multidrug CDR4 transporter, methotrexate resistance protein, opsin, ATPases and fumarate reductase. These play an important role in amino acid transport, toxin efflux and synthetic fungicide resistance [142], suggesting efflux of the agent or its metabolites. The authors’ data alone could not explain all apparent changes. The expression of a fungitoxic compound efflux protein (in particular, Major Facilitator Superfamily transporter mfsG) also increases in *Botrytis cinerea* when exposed to GSL-breakdown products: a study [143] has presented that mfsG-deficient lines of *B. cinerea* show an increased accumulation of fluorescein ITC, an increased susceptibility towards ITCs, and were also less virulent to GSL-containing plants. In vivo relevance is highlighted by the fact that the colonization of GSL-deficient (*cyp79B2/B3*) *A. thaliana* mutants results in a lesser increase in expression of mfsG than the colonization of wild-type *A. thaliana*.

### 3.6. Inhibition of Aflatoxin Biosynthesis

A few studies provided data on the effects of ITC exposure on mycotoxin biosynthetic genes’ expression. The detailed study of [70] has found that all tested doses of allyl ITC significantly inhibited the growth and AFB_1_ production of *Aspergillus flavus* in vitro as well as on stored maize. The inhibitory effect was found to be dose-dependent. This phenomenon is a very useful possible application of the ITCs for preservation of grains and bakery products, detailed later in Section 7.2. The authors tested the changes in expression of several genes of the 27 belonging to the aflatoxin biosynthetic cluster, as well as others. Interestingly, probably as a part of a general stress response, the upregulation of secondary metabolite biosynthesis genes, including those of the aflatoxin gene cluster was also observed during allyl ITC exposure. The genes that showed the highest upregulation included *meaB* and *laeA*. The gene *meaB* encodes a bZip protein eukaryotic environmental transcription factor which was shown to control infectious growth of *Fusarium oxysporum*. It is likely responsible for controlling nitrogen flow towards secondary metabolite biosynthesis. The genes *veA* and *laeA* are parts of the velvet complex, involved in light signal coordination: the deletion of *veA* prohibits aflatoxin biosynthesis as the main regulatory factor *aflR* is then inhibited. It is also essential for sclerotial formation and for survival under stress conditions. During allyl ITC exposure, a series of aflatoxin biosynthesis related genes were also upregulated versus β-tubulin as reference: *aflI*, *aflH*, *aflK*, *aflT*, *aflB* as well as the most important ones, *aflR* and *aflS* which form a dimer to activate the biosynthesis cluster. Upregulated global transcription factors include *nsdC*, *mtfA* and the downregulation of *fcr3* was observed. Cellular signaling and reception pathway genes showed mixed regulation upon allyl ITC exposure. Another study [89] came to similar conclusions when examining the inhibition of mycotoxin production by benzyl ITC in *Alternaria alternata*: production of alternariol monomethyl ether, alternariol, altenuene and tentoxin was inhibited during the treatment. These results suggest that the slower growth and the massive amount of resources that are consumed by oxidative defense more than offset the increased biosynthesis of the aflatoxin per cell in such a setting, rendering the net result a decrease in measurable aflatoxin amounts.

## 4. Quantitative Structure—Activity Relationship (QSAR) Data

A few studies were also carried out to obtain QSAR data on ITCs regarding antimicrobial (antifungal) activity. These studies began as early as the 1960s. The study by [51] tested various ITCs, including methyl, ethyl, isopropyl, 3-(methylsulfonyl)propyl, 4-(methylsulfonyl)butyl, 5-(methylthio)pentyl, benzyl, phenethyl, 4-methoxybenzyl ITC, as well as various synthetic ITCs like chlorinated aromatic ones on *Aspergillus niger*, *Penicillium cyclopium*, *Rhizopus oryzae* and other fungi. A 0.4–30 × potency was found compared to the reference allyl ITC; the highest activity was obtained with arylalkyl derivatives: phenethyl, 4-methoxybenzyl, and benzyl ITC. Clearly, these are significant differences. When [144] compared the antifungal potency of monosubstituted aromatic ITCs, the authors found that the para-substituted derivatives are highly fungicidal when compared to the control, but the ortho- and meta-substituted derivatives only possessed a fair activity.

The different reactivity of the various ITCs towards thiols may logically contribute to the difference of potency among various ITCs. The speed of the ITC—GSH conjugation was shown to strongly depend on the side chain [145]. In non-enzymatic reactions, 5-fold differences were found, while in enzymatic reactions (that possibly dominate in vivo conjugation), 100-fold differences were observed between the fastest and slowest reacting ITCs. Though reactivity correlates positively with a lower electron density at the reacting carbon atom of the ITC [13,146], we should note that this also changes penetration ability through lipid membranes as well as the polarity changes of proteins whose side-chains are modified. The latter might be behind the usually higher potency of aromatic ITCs and long side-chain ITCs compared to short side-chain ITCs, as detailed later. What is more, reactivity and hence efficacy may strongly vary depending on the experimental conditions as well. First of all, ITC reactivity is a function of pH. High pH pushes the ITC toward enhanced reactivity with thiols, alcohols and amines, as ITCs actually react with the dissociated form of these groups [13]. Nevertheless, the pH range usually found in organisms and their media in vitro usually enables the reaction to take place. These all result in an inability to easily predict antifungal activity for a given strain under particular conditions. In other words, as the proteome and the intracellular pH has variability, the strongest antifungal agents likely differ from strain to strain.

An example to this is the study of [109] who tested inhibition of soil-borne pathogens of wheat (*Gaeumannomyces graminis var. tritici*, *Rhizoctonia solani*, *Fusarium graminearum* and *Bipolaris sorokiniana*) in vitro by ITCs from *B. napus* (allyl ITC) and *B. juncea* (phenethyl ITC), added as plant tissues. Within each species, the different isolates showed different sensitivity to the tested agents. The same group of authors tested a series of ITCs against the same fungi in a subsequent study [34]: Four alkenyl aliphatic (methyl ITC, allyl ITC, butenyl ITC, pentenyl ITC) and two aromatic ITCs (benzyl and phenethyl ITC) were tested. Due to their lesser volatility, aromatic ITCs were less toxic when administered as vapor, but were more toxic to fungi when dissolved in the culture medium. *Gaeumannomyces* was the most sensitive, *Rhizoctonia* and *Fusarium* showed intermediate sensitivitiy, while *Bipolaris* and *Pythium* were more tolerant. The higher potency of less polar compounds was also described in [147]: nonyl, decyl, and dodecyl ITCs stimulated the germination of *Puccinia punctiformis* teliospores, while various naturally occurring, more polar ITCs (allyl ITC, benzyl ITC, phenethyl ITC) were inactive in the experimental setup. Such a pattern was not clearly found in the QSAR study of [52] on various ITCs against *C. albicans*. In the latter study, the methylthio-alkyl ITC erucin and methylsulfonyl-alkyl ITC sulphoraphane showed similar minimum inhibitory concentration (MIC) values to allyl ITC, but aromatic ITCs were either significantly more potent (benzyl ITC) or less active (phenyl ITC) than allyl ITC. The difference among strains regarding ITC sensitivity is perhaps best presented by the results in [80]. The authors tested the antimicrobial activity of an alkyl ITC homologue series from C_1_ to C_8_. Interestingly, while a bacterium, *Erwinia carotovora* showed a linear QSAR with being most sensitive to n-octyl ITC and least sensitive to methyl ITC, a fungus, *Rhizoctonia solani* was least sensitive to n-pentyl ITC, and a significantly higher potency was shown for both shorter and longer side chain ITCs. The authors concluded that steric hindrance might be in the background. In [37], it was shown that in case of long-chain methylthioalkyl ITCs, oxidized forms (such as 9-(methylsulfinyl)nonyl ITC) had better activity than non-oxidized forms. The authors suspect that this occurred possibly because of increased polarity. A logical biological variable that strongly influences the uptake of semi-lipophilic or lipophilic toxins is the structure of the cell barriers: the type of cell wall (if present) and the structure of membrane(s)—probably, this is why [37] found different sensitivity of Gram^+^ and Gram^−^ bacteria towards short-, and long-chain methylthioalkyl and methylsulfonylalkyl ITCs.

The ITCs’ side chains can be relatively large compared to the -N=C=S group alone. This results in various water solubility and volatility parameters within the group of GSL-derived ITCs (Figure 1). Therefore, in the case of vapor phase applications, higher water solubility logically enhances activity. An example is shown in the study of [148] who found that *Cladosporium cladosporioides*, *Aspergillus niger* and *Penicillium citrinum* are better inhibited by allyl ITC in an environment with higher relative humidity. This explains the widespread use of allyl ITC in food preservation and biofumigation applications, detailed in Section 6 and Section 7.

Altogether, the size and chemical nature of the ITC side chain predictably influences polarity, volatility and reactivity of the compounds, but still, no one-size-fits-all rule can be set to predict the antifungal potency in liquid-phase experiments. The aromatic and more apolar ITCs are usually more potent than short-chain aliphatic ones, but there are exceptions. Reactivity and bioactivity also strongly depend on environmental conditions, first of all, pH. On the other hand, vapor-phase applications require good volatility, which increases with small molecule size, which in turn leads to better water solubility as well.

## 5. Synergistic Activity

A few papers describe synergistic activity between different ITCs, or between an ITC and a non-ITC antifungal agent. Plant defense systems usually contain mixtures of similar compounds that frequently act as synergistic agents against pathogens; the case of ITCs seems to be no different.

Papers dealing with synergy between GSL-derived compounds include the study of [53] who have found synergistic antifungal activity between allyl and ethyl ITC against infection of apples by *Penicillium expansum* and *Botrytis cinerea*. The conidial germination and mycelial growth were both inhibited by the combination. The antifungal synergy between allyl and phenethyl ITCs and nitriles was studied by [93] against *Candida albicans*, *Penicillium notatum* and *Aspergillus niger*. The authors used a mixture of three components and presented a synergistic action. Allyl and phenethyl ITC from horseradish essential oil also showed synergistic antifungal activity against *C. albicans* in [56].

Regarding synergy between ITCs and non-GSL-derived compounds, a few studies were published to date. The paper by [54] preseted synergistic antifungal effects of vapor phase ITC combinations against *Penicillium notatum* for the following compounds pairs: sulfur dioxide/allyl ITC, allyl ITC/cinnamaldehyde. Sulforaphane and paraben showed synergistic antifungal activity against *Candida albicans* and *Aspergillus niger*, likely resulting from cell membrane damage and cell leaking [149]. Benzyl ITC increased the fungicidal activity of amphotericin B via vacuole disruption in the model of *Saccharomyces cerevisiae* [150].

## 6. Biofumigation, Inhibition of Plant Pathogenesis

Several studies used ITCs, or plant materials that release ITCs as biofumigating agents. The potency of the application of ITC containing plants is best presented by [151], concluding that *Brassicaceae* biofumigation can be a viable substitution for chemical biofumigation treatments on the long-term, after assessing results of a 13-year study in field potato production.

### 6.1. Plant Protection Studies

Plant greens are typically used as soil amendments in studies aiming to reduce pathogenesis of a crop. The most frequently used species are young plants of *Brassica juncea*, *B. napus*, *B. rapa*, *B. oleracea*, *B. carinata* and *Sinapis alba*. The results mainly include disease suppression of the plant of interest in several crop-pathogen pairs [48,49,74,75,104,113,152,153,154,155,156,157,158,159,160,161]. The studies include in vitro, pot and field studies as well, summarized in Table S3.

There are a few reports on spray applications as well. Powdery mildew fungi *Erysiphe betae* and *Erysiphe cichoracearum* were controlled by spraying a dispersion of *Brassicaceae* meal in vegetable or mineral oils on infected leaves of sugar beet or cucumber, resulting in a significant decrease of the infected leaf area [162]. Olive leaf spot caused by *Fusicladium oleagineum* was inhibited by a dispersion of *Brassicaceae* meal in vegetable oil in vitro and in field experiments [163]. Allyl ITC releasing *Brassica carinata*-based emulsion products (containing 1.5% and 2% *B. carinata* oil) successfully inhibited powdery mildew on melon in a field study, showing the same efficacy as the standard fungicide Topas 10EC used in a 0.25 mL L^−1^ dose [164].

Pure compounds used for biofumigation include methyl ITC (often released by incorporating metham sodium into the soil), but direct incorporation of other volatile ITCs, typically allyl ITC is also described. The advantage of this approach is being chemically more defined, of course, at the cost of the loss of long-term microbial growth-promoting effects, as detailed later on. With this approach, effective prevention of fungal diseases was successful in several instances [47,165,166,167,168]. Some additional details are summarized in Table S3.

### 6.2. Parameters Influencing Potency

The soil amendment’s efficacy against *Verticillium dahliae* was found to depend on temperature and water content of the soil: at 2 °C, in wet soil, the agent was more efficient [169]. Allyl ITC release from seed meals was also shown to strongly depend on some soil parameters in [114]. The authors found that increased water potential (−40 kPa) and higher temperatures (30 °C) facilitates ITC release, whereas a saturated environment (0 kPa) inhibits it. Another recent study by [107] confirmed the impact of water availability: when studying the inhibition of the chilli pepper pathogen *Verticillium dahliae* by mustard seed meal, rates of GSL degradation were found to be the highest in water-saturated soil, resulting in the highest efficacy against *V. dahliae* in the experiment.

### 6.3. Contribution of Isothiocyanates and Organic Matter to Soil Microbiome Changes during Biofumigation

Several experiments have examined the effects of ITCs, or the incorporation of ITCs and ITC-containing plant materials into soil on soil microbial communities. This is thought to be the theoretical basis of biofumigation studies.

Although it would be an attractive interpretation, the plant material should not be considered an inert vehicle that delivers the bioactive, antifungal ITCs into the soil. Long-term effects are to a large extent caused by changes in soil microbiome, and these changes are in part triggered by nutrient addition in the form of plant material. This is underlined by the fact that, despite all the preconceptions, the effects on fungi were shown to be somewhat independent of GSL content in several papers [170,171,172,173] suggesting that the nutrient surplus alone can explain the changes in the microbiome that ultimately leads to inhibition of plant pathogenesis. In [174,175,176], plant material incorporation resulted in an increase in total fungal colony count and soil microbial activity.

Of course, this does not mean that the ITCs do not contribute any activity, but long-lasting effects are not always delivered. While the chemical reactivity of ITCs [13] enables them to have high bioactivities, their persistance in soils is low for the same reason. ITCs react readily with thiols and amines in organic matter via chemical reactions [51,146], which results in reduced antifungal efficacy for ITCs when high amount of organic matter is present, as shown by [177]. This is very similar to the efficacy difference found between nucleophile-poor and nucleophile-rich media in [37]. Therefore, it is unsurprising that ITC detection from soil was inconsistent in the study of [178], who could not detect any ITCs in soils after 12 days. Methyl ITC fumigation (administered as metham sodium) causes a short-term transient reduction of total fungal CFUs in soil, and subsequent recovery takes about 40 days, as shown by [179]. The impact of ITC on *R. solani* growth also seemed to be short-term only in [180], and no ITC could be detected from the soil after 7 days. These authors also concluded that long-term suppressive effects are likely caused by changes in microbial composition and suppression of the pathogen.

Therefore, it is not that surprising that under some conditions, the application of green manures of *Brassica* species can support the saprophytic and other activities of pathogens, resulting in negative study results. An example is [181] who reported that the amendment caused increased activity of *R. solani* resulting in an increase of damping-off in canola [181], despite that pure ITCs inhibit growth of the fungus, and amendments significantly reduce hyphal growth of *Rhizoctonia solani* [59].

Moreover, in some instances, an increased pathogen population was found as the result of amendment, as in the study of [178]. In this case, the amendment also failed to consistently lower incidence of damping-off and *Fusarium* wilt on watermelon. Other studies that report failed inhibition of plant pathogenesis include [182]. The actual reason for such outcomes may vary.

### 6.4. Group-Level Changes in the Soil Microbial Community after Biofumigation

Several studies have shown that ITCs alter fungal microbiome compositions and the abundance of fungi. The effects are very variable overall, suggesting that individual sensitivities of the fungal strains, and composition of the initial microbiome likely have a fundamental influence on such phenomena. Although the fungal community is usually significantly disturbed by these treatments (detailed later), an increase in overall fungal abundance was found in several studies [183,184,185,186]. In [183], kinetics were similar for the various treatments, suggesting that long-term disease control is rather a consequence of changes in microbial composition by addition of nutrients, rather than direct antifungal activity. In one study, a difference among different *Brassicaceae* was also found [184], suggesting contribution of ITCs to microbiome changes.

Studies showing a decrease in fungal populations (and diversity as detailed later on) are more frequent. This suggests that effects on the microbiome is in part the result of Brassicaceae-specific compounds. In a study by [187], addition of the leaf residues from *Brassica rapa*, *B. napus* and *B. juncea* to soils suppressed the plant pathogen *Rhizoctonia solani*. Importantly, this suppression correlated to the increased Actinomycetes/fungi ratio in the soils, but not the in vitro antifungal activity of the plant extracts. Fungi were expressed as total CFU and quantified using selective growth media. Effects of amendments on total CFU depended on the experiment site as well as the amendment species and ranged from 4-fold decrease to about 1.8-fold increases. In another paper, the introduction of camelina (*Camelina sativa*) into a wheat monoculture system decreased the fungal abundance as shown by phospholipid fatty acid analysis [188].

A study with pure ITCs was also conducted by [189] who assessed the long-term alteration of the microbial community of a sandy loam soil using synthetic biofumigants in a microcosm experiment. The tested agents were methyl bromide and various chlorinated agents as well as methyl ITC. In contrast to bacteria, the fungi (including mycorrhiza) were not significantly altered by the methyl ITC treatment. Soils treated with other agents showed lower abundance of fungi; overall, methyl ITC had the least effect on soil fungi when compared to the other tested agents. In a microcosm pot study of [190], the effects of plant-material-based biofumigation and that with pure compounds was compared. Incorporation of *Brassica* residues without added myrosinase increased fungal biomass. To a lesser extent, the same treatment with added myrosinase resulted in the same. The latter treatment resulted in complete GSL hydrolysis. However, treatment with the synthetic soil fumigant metham sodium did, but phenethyl ITC did not result in a temporary decrease of fungal abundance.

### 6.5. Functional Studies

The study of [191] examined the effects of *Limnanthes alba* (Limnanthaceae, Brassicales) amendments on soil microbes. The activity of the soil microbiota was assessed by enzymatic stuides and Biolog EcoPlates™. Amendments increased the basal respiration rate and stimulated some enzyme activities. Moreover, the different treatments resulted in different carbon source utilization patterns. The authors also measured the abundance of several fungal genera belonging to the Ascomycota, as detailed in the next section. During a methyl ITC biofumigation study in a sandy loam soil [189], acid phosphatase, aryl-sulfatase and dehydrogenase activity was significantly altered, compared to the untreated soil. A microcosm-study by [190] found that *Brassica* residues with or without added myrosinase stimulated soil respiration, and microbial activity. The plant material without myrosinase was found to be more effective. On the other hand, treatment with metham sodium or phenethyl ITC resulted in a temporary decrease. This suggest an antagonism between the ITCs and the organic matter in the experimental setup.

### 6.6. Pattern Change Studies by Gel Separation Techniques

The soil microbiome pattern changes caused by a transgenic *A. thaliana* with sorghum CYP79A1 was tested in the study of [192], in which the plants were grown under a ^13^CO_2_-enriched atmosphere. The used *A. thaliana* line produces p-OH-benzyl GSL and another unidentified GSL as well as those produced by the wild type. ^13^C CO_2_ was used to verify the usage of root exudates by microbes. A denaturing gradient gel electrophoresis (DGGE) analysis showed that fungi were obviously affected by GSL content. In ^13^C-labeled populations, the fungal community was found to be affected by the production of exogenous GSLs. For example, the fungal parasite *Syncephalis depressa* (*Zoopagomycotina*) was only found in the root microbial community of transgenic plants. What is more, the species was shown to be able to utilize ^13^C-labelled plant exudates. The study of [190] has found that incorporation of *Brassica* residues with or without myrosinase into soils, as well as fumigation with metham sodium or phenethyl ITC created short-term changes in the fungal community. The composition of the fungal microbiome was assessed by DGGE, followed by sequencing of clone libraries. Several fungi, including homologues to potential plant pathogens (*Fusarium*, *Nectria*, and *Cladosporium*, *Phaeoacremonium*) were present in all treaments, with the metham sodium treated soils being the only exception. The same treatment resulted in the dominance of an *Eurotium* strain, a *Hypocreales* isolate and a *Fusarium proliferatum* strain. Plant material incorporation resulted in much less alterations, but a few DGGE bands exclusively appeared in treatments with plants, without myrosinase (e.g., that of *Pyrenochaeta*). A biofumigation study with defatted *Brassica* seed meals has studied the effects on the fungal community in a pot experiment [193]. The fungal community was sensitive to organic matter (sunflower seed meal), synthetic ITC (metam-sodium fumigant) as well as biofumigant (*B. carinata* seed meal). Importantly, the ITC and biofumigant treatments clustered together based on their fungal 18S rRNA DGGE profiles, suggesting the effects are in part due to the ITCs themselves. The biofumigant treatments also reduced the incidence of *Fusarium solani* on tomato plants after 60 days, Rhizoctonia solani was only found in non-treated controls. Yet, the overall microbial diversity did not significantly decrease after treatment with the biofumigant or ITC. The fungal community was also changed by soil amendments from *Brassica juncea* or *Raphanus sativus* according to [194], who assessed alterations by 18S rRNA and ITS DGGE profiles. Different combinations of anaerobic soil disinfestation and mustard seed meal amendment changed microbial composition of soils in [195]. The mustard seed meal amendment was shown to control apple replant pathogens: DNA of *Pythium ultimum* and *Rhizoctonia solani* in roots was reduced by treatments vs. controls, but this did not result in reduced infection rates. *ANOSIM* of *t-RFLP* patterns of fungal sequences also showed differences between control and treatments.

### 6.7. Pattern Change Studies by Sequencing

Despite the changes in fungal abundance were described in many instances, a real insight on the impact of ITCs can only be obtained via detailed analysis of the underlying community with next generation sequencing methods. Several studies have reported reduction in overall fungal diversity and/or reduction of given genera during treatment with ITCs or ITC-containing plant materials. A comparative microcosm experiment by [196] tested the effects of seed meals of various plant species on the fungal and bacterial community in soil. The experiment was carefully designed to include mustard (a seed with fixed oil and GSLs), and flax (a seed with fixed oil but no GSLs) and sorghum (contains neither) as controls. Importantly, a distinct separation was observed by amendment type. Mustard induced large increases in the abundance of bacterial taxa associated with fungal disease suppression (e.g., *Bacillus*, *Pseudomonas*, and *Streptomyces* spp.). The phylotype richness of fungi decreased by >60% at the same time: all detected species (incl. *Alternaria*, *Fusarium*) showed reduced abundance except *Retroconis*. Other seeds (flax, sorghum) had less pronounced, mixed effects on fungi. The rapid changes were not followed by recovery: the effects persisted throughout the examination period of 28 days. But as recovery was only observed at 60 days by [190], this might have been the result of early termination of the experiment. A dramatic decrease (about ~85% reduction) in fungal populations upon ITC treatment was also observed in the interesting approach of [197], who used a flax-seed soil amendment but supplemented it with added ITCs (allyl, butyl, phenyl, and benzyl ITC). In this work, community changes were examined using qPCR and tag-pyrosequencing with 454, using fungal ITS sequences. Community changes showed transient and long-term changes as well. In all ITC treatments, an increase in *Ascobolus* and *Chaetomium*, a transient rise in *Retroconis*, the relative tolerance of *Fusarium* as well as a decrease in fungal diversity (decrease in *Rhizoctonia*, *Thanatephorus*, *Mortierella*, *Monographella*, *Laetisaria*) was observed. Similar results were obtained by [198], who treated soils with rapeseed-derived ITCs (goitrin and 3-butenyl ITC being the major components): despite being highly diverse at initial conditions, Ascomycota and Zygomycota became almost completely extinct in treated soils. A significant reduction in the saprophytic *Mortierella* was observed. Meanwhile, Basidiomycota prevailed, *Trichosporon* being the most abundant genus in treated soils. Less pronounced effects were found in the study by [199], but the results clearly show the impact of plant GSLs on soil fungal communities. The study tested the changes of the microbiome of *Brassica napus* L. (subsp. *oleifera* cv. Tenor) in response to belowground herbivory by *Delia radicum*. The herbivory results in an altered GSL pattern, in particular, an increase in glucobrassicin and neoglucobrassicin as well as a reduction in glucoerucin, gluconapin, progoitrin compared to controls. This influenced soil and rhizoshpere fungi to some extent: a more intense reduction of Chytridomycota and Blastocladiomycota in the rhizosphere was observed compared to controls, however, the effect of sampling time alone had a significant effect. The root compartment changes were similar. A much more significant effect on diversity and composition of bacteria was shown in the same experiment. In the seed meal amended soil study of [183], *Fusarium* and *Chaetomium* accounted for more than 50% of operational taxonomic units (OTUs). Treatments promoted *Fusarium*, *Chaetomium* and *Humicola* proliferation in the mesocosms, but still reduced overall fungal diversity. The changes were relatively persistent over time. Interestingly, GSL-containing *Camelina* amendment resulted in a fungal community similar to that seen in control and that obtained by amendment with flax or *Jatropha* (*Euphorbiaceae*), but quite distinct from the one that resulted from amendment by wheat straw.

Only one study with detailed microbial community composition data has reported an increase in fungal abundance after *Brassica* amendments of soils. Fungal abundance was increased by all *Brassica* seed meal treatments in the study of [200], regardless of GSL content. The treatments included low GSL *Camelina sativa*, high GSL *Brassica juncea*, a 1:1 mixture of high GSL *Sinapis alba* and high GSL *B. juncea*. The abundance of fungi significantly increased as a result to all soil amendments at day 25, followed by a subsequent decrease during cultivation of chili pepper in the same soils (35 days). The treatments also resulted in different species composition and incidence of *Fusarium* wilt. The *B. juncea* and *B. juncea* + *S. alba* treatment resulted in increase of *Chaetomium*, inhibition of *Hypocreales*, and also negatively correlated with disease incidence. *C. sativa* resulted in an increase in Mortierellales. Control treatments were rich in Gibbelulopsis and Pleosporales. Interestingly, the *Brassica* seed meals had more pronounced effects than the chili plant itself, as no significant changes were seen in that phase compared to the beginning of plantation [200].

Studies with pure compounds also show a significant decrease in fungal diversity. For example, allyl ITC biofumigation resulted in community changes in soils of tomato according to [201]. Ascomycota, Zygomycota, Basidiomycota and Chytridiomycota had the highest relative abundances in decreasing order. After allyl ITC treatment, the relative abundance of Ascomycota and Sordariales decreased significantly, while the relative abundance of Microascales, Hypocreales, Eurotiales and Onygenales increased significantly, Mortierellales showed only a temporary increase. In contrast to bacterial communitites, fungal communities did not recover by the end of the 122-day treatment. In fact, Principal Coordinates Analysis (PCoA) could easily distinguish treatments’ effects on fungal, but not bacterial communities. Upon treatment, the abundance of *Pseudallescheria*, *Acremonium*, *Corynascus*, *Remersonia*, *Kernia*, *Aspergillus* and that of an unidentified fungal group increased. *Fusarium* showed an initial increase in abundance, followed by a reduction in high-dose treatments by the end of the study.

Overall, the community changes caused by Brassicaceae amendments seem to be difficult to predict. This is likely due to the various chemistry of the incorporated plant materials, the variability of the initial community compositions, and many other, untested factors. However, it seems likely that the plant materials themselves generate a shift in microbial composition that is usually favorable for prevention of plant pathogenesis. In several instances, the reduction of fungal activity and diversity was described both in pure ITC studies and plant amendment studies.

### 6.8. Combinations with Other Biocontrol Agents

The biofumigation agent plant amendments are often combined with biocontrol fungi, and synergistic activity is found on various crops, in many instances [173,202,203,204,205]. The mechanism behind this synergy might be the higher tolerance of the biocontrol fungus *Trichoderma harzianum* against *Brassica* soil amendment decomposition products compared to phytopathogens, as shown in several papers [173,202,203]. Some additional details are given in Table S4.

### 6.9. Effect on Mycorrhizae and Isothiocyanate-Mediated Allelopathic Activity

There are a few studies on the lack of activity on mycorrhizae: no negative effect on mycorrhizal colonization of *Zea mays* crops was found by addition of B. napus residues [172]. Winter crop forage radish (*Raphanus sativus var. longipinnatus*) also lacked any effect in *Zea* in a multi-year study [206].

In contrast, ITC release from *Brassicaceae* plants was shown to confer allelopathic effects via effects on ectomycorrhizal fungus (EMF) colonization [58]. Theoretically, as Brassicaceae-plants usually do not harbor mycorrhizae [2], but most other plant species do, reduction of the mycorrhizal colonization of other species can give these plants a competitive edge [207]. Of other GSL producing plant families, Capparaceae and Phytolaccaceae were diagnosed as non-mycorrhizal, while in the Resedaceae, both arbuscular mycorrhizal species and non-mycorrhizal species were found. The reason for a non-mycorrhizal character might not be a consequence of GSLs as a whole group. Rather, this preference might be attributed to a GSL type and/or a given set of individual GSLs. E. g. glucobrassicanapin (alkenyl GSL) is present in Brassicaceae but not in other GSL-containing families [2].

A well-studied example of allelopathy is that of the invasive *Alliaria petiolata*, which was reviewed in [208,209]. A field comparison of *A. petiolata* patches and control areas revealed that root tip biomass was lower in invaded soils, the effect was most prominent at the immediate neighborhood of the patches, and in conifer-dominated forests [210]. The overall fungal community and AMF were both significantly changed by concentrations of GSLs in roots of *Alliaria petiolata* [211]. The allelopathic effects only affected mycorrhizal plant communities in a manipulative 11-year field experiment [212]. The *Alliaria petiolata* amendments have effects similar to that found in case of fungicide application according to [213].

By studying competition among *Brassica nigra* genotypes (low sinigrin, high sinigrin, heterospecific), [207] have shown that the dynamic interaction and competition between these genotypes is mediated by the effects of sinigrin on arbuscular mycorrhizal fungi. It might seem somewhat paradoxical that some plants secrete sufficient amounts of ITCs (or their precursors) to the soil to enable achieving an allelopathic effect, while at the same time incorporation of GSL-containing soil amendments usually result in no measurable inhibition of mycorrhizae. The background behind this issue can be qualitative or quantitative reasons (GSL pattern), possible differences between organs [214], not to mention the possible differences between plant-mediated and microbe-mediated decomposition of GSLs [4,215].

### 6.10. Phytotoxicity

Logically, plants—especially non-*Brassicaceae* plants—can also be sensitive to ITCs; the background mechanisms are likely the same as in the case of antifungal activities. It is noteworthy that high doses of ITCs or soil amendments containing them can result not only in disease suppression but also in phytotoxicity, as shown in several studies [159,161,170,216]. In these studies, amendments were typically *Brassica* plants or seed meals.

## 7. Preservation and Other Applications

There are several applications that show efficient preservation of bread, bakery products as well as fruits and vegetables in the post-harvest phase. Frequently cited advantages include no residual off-flavor, no chemical changes, low required amounts and good volatility. In this section, the scientific literature dealing with such applications is summarized. In many instances, the inhibition of growth and aflatoxin biosynthesis are presented in the same study. The previously mentioned works of [70] on transcriptomics suggest that the decrease of aflatoxin amounts is due to the reduced growth, which more than offsets defensive biosynthesis of such compounds. Nevertheless, data show that ITC vapor is a very efficient agent to deal with food spoilage.

### 7.1. Grains and Oil Seeds

Several studies were carried out on effects of ITCs increasing shelf-life or microbial stability of stored grain products. In many instances, the prevention of microbial growth was presented, but several articles also show the inhibition of fungal toxin concentration growth as well. The technique was found to be suitable for long-term storage. A study by [217] tested inhibition of a storage pathogen of soybean (*Aspergillus glaucus*) by ITCs of mustard essential oil. Though complete eradication was not achieved, significant efficacy was shown: the treatment resulted in a slowdown of fungal growth, assessed by CFU number and free fatty acid content, which positively correlated. As much as a 1000-fold CFU and 6-fold free fatty acid (FFA) content increase was observed in non-treated samples compared with the fumigated ones, after 4.5 months of storage. Additional fungi inhibited by ITCs on grains include *Aspergillus flavus* [70,218,219], *Aspergillus parasiticus* [219,220,221,222,223], *Penicillium verrucosum* [219,224], *Fusarium verticilloides* [45,223], *Fusarium graminearum* [106], *Alternaria alternata* and *Gibberella zeae* [45]. The above experiments typically used allyl ITC vapor as the bioactive agent. The experiments were carried out on various widely used grains, including maize [45,70,219,225,226], peanuts [218,221], Brazil nuts [222], wheat [106,219] and barley [219,224]. Several studies also showed inhibition of the production of one or more aflatoxin variants [45,70,219,221,222,223,226], ochratoxin [219], *tricothecene HT-2 and zearalenone* [45]. In a few instances, several variants were quantified [45,70,223], including aflatoxins B_1_, B_2_, G_1_ and G_2_, fumosinins B_1_, B_2_. The study of [70] also supplied very interesting results on the transcriptomic response of the fungi and is dealt with in detail in Section 3.3.

### 7.2. Bread and Bakery-Products

Several studies have shown efficient inhibition of fungal growth on bread products by ITCs, applied in the form of e.g., modified atmosphere packaging systems which often resulted in a meaningful increase of product shelf-life. In a few studies, the comparative effectiveness compared to other plant essential oils, including monoterpene or phenylpropanoid-based essential oils was also investigated [76,227]. In both of these cases, ITC-containing essential oils were shown to be the most potent agents. In [76], mustard oil was shown to reduce the growth of *Penicillium commune*, *P. roqueforti*, *Aspergillus flavus* and *Endomyces fibuliger* on bread, at MIC values 1.8–3.5 μg mL^−1^ gas phase allyl ITC. The activity against these frequent food-spoilage fungi of bakery products was fungistatic or fungicide depending on the concentration. In [227], food spoilage fungi on rye-bread were successfully inhibited by allyl ITC and other plant volatiles. Other studies showed successful application of allyl ITC vapor on chilled pizza against *Penicillium nordicum* [77], on Pita bread against *Penicillium verrucosum* [228], and on pizza crust against *Aspergillus parasiticus* [219,229].

Aflatoxin-related studies include those of [78,105] who have shown that mustard flour or its ITCs (vapor phase allyl ITC, benzyl ITC, phenyl ITC) inhibit not only the growth of *Aspergillus parasiticus* on bread-like products, including loaf bread and Italian piadina, but also the production of aflatoxins B_1_, B_2_, G_1_ and G_2_. The authors observed the complete loss of viability (fungicide activity) at 50 mg L^−1^. The same authors also showed that patulin production by *Penicillium expansum* on wheat tortillas was efficiently inhibited by allyl ITC alone or mustard flour [230]. The production of aflatoxin was also shown to be inhibited [228,229].

### 7.3. Dairy Products

In [220], allyl ITC alone or added as mustard meal inhibited growth of *Penicillium digitatum* and *Aspergillus parasiticus* in sliced mozzarella for 60 days, while shelf life of controls was 19-41 days, depending on the fungus and container. The treatment also inhibited production of aflatoxin B_1_. Allyl ITC was also found to be useful against cheese spoilage fungi in [231].

### 7.4. Post-Harvest Preservation of Fruits

Several studies were published that presented the successful inhibition of post-harvest quality loss of fruits. Again, the most widely used agent is vapor phase allyl ITC, however, some also used natural ITC mixtures with phenethyl ITC and phenyl ITC being the principal components [79], benzyl ITC [90,232] or phenethyl ITC [94]. Successful attempts to increase shelf-life were reported in pears [94,233], raspberries (*Rubus idaeus*) [234], bell peppers [79], melon [232], tomato fruits [90,235,236], strawberries [69,237]. The inhibition of *Penicillium expansum* [233], rot-causing *Alternaria alternata* [79,90,94,232], *Botrytis cinerea* [69,237], *Geotrichum candidum* and *Fusarium oxysporum* [235] was reported.

What is more, in several instances, reduced or inhibited loss of quality in fruits (e.g., general appearance) was accompanied by no changes in other organoleptically important quality parameters. Total soluble solids and firmness were not significantly influenced by ITCs [79,90,232,235]. This was also true for several other measured parameters in tomato, such as respiration rate, acidity, ethylene production [90], ascorbic acid content and CIELAB color [235], or, for phenolic content and antioxidant capacity for strawberry [69,237]. Though the authors of [90] concluded that benzyl ITC had no significant effect on the physiology of tomato fruits, a more in-depth study by [237] showed an increased amount of asparagine, ascorbate, and ITC-adducts in strawberries upon allyl ITC treatment. The authors also describe a transient glutathione depletion phenomenon. Also of significance is the fact that the benzyl ITC treatment also significantly decreased alternariol, alternariol methyl ether, altenuene, tentoxin production by *Alternaria alternata* on pears [94]. Altogether, though ITCs likely impact the metabolism of the plants in the post-harvest phase, this can still result in efficient protection against post-harvest decay without apparent changes.

### 7.5. Other Products

A few studies on miscellaneous food as well as some non-edible products were also published. Benzyl ITC and allyl ITC was compared in vapor phase for food preservation in [238]. A strong inhibitory activity against fungi and yeasts was found, allyl ITC being more effective against *Aspergillus sojae* and *Penicillium expansum* on rice cakes. Spoilage in fermented cucumber was also successfully inhibited by allyl ITC [239]. The effect was attributed to inhibition of lactic acid metabolizing yeasts *Pichia manshurica* and *Issatchenkia occidentalis*, which results in subsequent bacterial growth. We note that this application closely resembles the traditional use in Central Europe, where *Armoracia rusticana* roots of high ITC content are used for preservation of pickles [240].

In [241], a rocket essential oil (mainly consisting of erucin)—zinc sulphate combination was shown to inhibit growth of fungi *Fusarium oxysporum* and *Trichoderma* viride on old manuscripts. In [242], a β-Cyclodextrin—allyl ITC formulation decreased wood mass loss when exposed to brown and white rot fungi.

## 8. Conclusions

The wide array of publications on their antifungal activity leaves no doubt that the ITCs, the main decomposition products derived from glucosinolates, are efficient agents against a wide range of fungal strains. Many plant pathogens, human pathogens as well as other fungi were shown to be inhibited in vitro by these agents. The chemical variability of the glucosinolate side-chains results in various ITCs with different antifungal potency, polarity, volatility and toxicokinetic properties. One can conclude from the results of the quantitative structure-activity relationship studies though, that there is no one-size-fits-all rule to predict antifungal activity. In general, less polar compounds are usually more potent in solution, but lag behind more volatile compounds with small molecule size in vapor-phase applications. Recent research on the background mechanisms of the antifungal activity has probably just scratched the surface, but the main biochemical targets seem to be directly related to the chemical reactivity: the antioxidant machinery is one of the well identified targets. Excellent studies on transcriptomics show general stress responses and, importantly, the inhibition of the production of fungal toxins is shown in many real-life applications as well.

ITCs’ natural origin and biodegradability makes them a good candidate for a wide range of possible applications, which is shown in an impressive number of papers. This range of applications is well extended by some widely available ITCs, such as allyl isothiocyanate, being relatively volatile. A major part of these applications correspond to biofumigation. During biofumigation, a soil amendment is prepared from plant species that contain glucosinolates which are allowed to break down to ITCs after incorporation into the soil. A detailed insight on microbiome changes is now available in the literature, again, showing that there are complex mechanisms behind the technology. The plant material should not be considered an inert material containing ITCs. In fact, the plant material as a nutrient source can cause long-lasting beneficial effects on the soil microbiome, despite ITCs are usually only detectable in the short-term. More importantly, several studies have shown that biofumigation is a useful technology to reduce fungal pathogenesis of a variety of crops. Long-term studies with promising results are also available, and the effects are delivered usually without apparent side-effects to the plants. Additional applications include inhibition of fungal growth, pathogenesis and/or toxin production in a variety of stored plants, grains, inhibition of disease on fruits in the post-harvest phase as well as increasing the shelf-life of different food products. What is more, as several studies demonstrated, the decrease in decay is frequently accompanied by the lack of measurable changes in various quality characteristics. More in-depth studies will be required in this field to assess concerns regarding safety, but due to the advantages of ITCs, it is likely that there will be a significant increase in demand and interest for such applications.

## Supplementary Materials

The following are available online at https://www.mdpi.com/article/10.3390/jof7070539/s1, Supplementary File S1: The search query used in Scopus searches, Supplementary Table S1: CAS identifiers of the compounds searched in SciFinder, Supplementary Table S2: A review of direct effects of non-standardized, isothiocyanate containing plant extracts in in vitro models using in-medium or vapor exposure, Supplementary Table S3: Studies including in vitro, pot and field experiments with ITCs or glucosinolate containing plant biofumigants, Supplementary Table S4. Review of plant protection studies combining glucosinolate containing plant amendments and biocontrol bacterial or fungal strains.
