# Supplementary material for: Effects of Glucosinolate-Derived Isothiocyanates on Fungi: A Comprehensive Review on Direct Effects, Mechanisms, Structure-Activity Relationship Data and Possible Agricultural Applications
Source: J Fungi (Basel). 2021 Jul 6;7(7):539. doi: 10.3390/jof7070539 (PMC8305656; doi:10.3390/jof7070539)
Supplement: Supplementary file 1 [file jof-07-00539-s001.zip › Supplementary_material_1.pdf]

The current review is based on the articles found from the searches in Scopus and SciFinder, the current review aims to be rather exhaustive in coverage regarding the literature. Therefore, all known GSLs, their respective ITCs were searched in one of the platforms, either as name in abstracts / titles in Scopus, or as CAS entities in SciFinder. The bibliographies from the two platforms were merged and filtered to remove duplicates in R [1]. The search queries and the search CAS numbers were added as supplementary materials.

#Scopus search query
(TITLE-ABS-KEY (fung* AND (glucosinolat* OR isothiocyanat* OR myrosinase))) OR ((TITLE-ABS-KEY (fung*) AND TITLE-ABS-KEY (("1-Acetylglucobrassicin" OR dehydroerucin OR epiprogoitrin OR glucoalyssin OR glucoarabin OR glucoaubrietin OR glucobarbarin OR glucobenzosisymbrin OR glucobenzsisaustricin OR glucoberteroin OR glucobrassicanapin OR glucobrassicin)))) OR ((TITLE-ABS-KEY (fung*) AND TITLE-ABS-KEY ((glucobrassicin-1-sulfate OR glucocamelinin OR glucocapangulin; AND glucopangulin OR glucocapparin AND b OR glucocappasalin OR glucocheirolin OR glucocleomin OR glucocochlearin OR glucoconringiin OR glucoerucin OR glucoerypestrin)))) OR ((TITLE-ABS-KEY (fung*) AND TITLE-ABS-KEY ((glucoerysolin OR glucohesperin OR glucohirsutin OR glucoibarin OR glucoiberin OR glucoiberverin OR glucojiabutin OR glucolepidiin OR glucolepigramin OR glucolesquerellin OR glucolimnanthin OR glucomalcomiin OR glucomatronalin)))) OR ((TITLE-ABS-KEY (fung*) AND TITLE-ABS-KEY ((gluconapin OR gluconapoleiferin OR gluconasturtiin OR gluconorcappasalin OR glucoputranjivin OR glucoraphanin OR glucoraphenin OR glucosinalbin OR glucosisaustricin OR glucosisymbrin OR glucotropaeolin OR glucoviorylin)))) OR ((TITLE-ABS-KEY (fung*) AND TITLE-ABS-KEY (("4-Hydroxyglucobrassicin" OR 4-methoxyglucobrassicin OR napoleiferin OR neoglucobrassicin OR progoitrin OR sinalbin OR sinigrin))))

1. R Core Team *R: A Language and Environment for Statistical Computing.*; Vienna, Austria, 2020;
