# Supplementary material for: Effects of Glucosinolate-Derived Isothiocyanates on Fungi: A Comprehensive Review on Direct Effects, Mechanisms, Structure-Activity Relationship Data and Possible Agricultural Applications
Source: J Fungi (Basel). 2021 Jul 6;7(7):539. doi: 10.3390/jof7070539 (PMC8305656; doi:10.3390/jof7070539)
Supplement: Supplementary file 1 [file jof-07-00539-s001.zip › Supplementary_table_2.pdf]

Supplementary table 2. A review of direct effects of non-standardized, isothiocyanate containing plant extracts in in vitro models using in-medium or vapor exposure. The exact concentration of isothiocyanates or glucosinolates were not indicated in the manuscripts.

Source	Fungal strains	Fungus functions	Activity	Ref.
Aethionema saxatile	*Aspergillus quadrilineatus*	PP	MG	[1]
Brassica campestris	*Chrysosporium indicum, Trichophyton rubrum, Trichophyton simii*	HR, ENV	MG	[2]
Brassica carinata	*Fusarium circinatum*	PP	MG	[3]
Brassica carinata	*Alternaria dauci, Alternaria radicina, Ascochyta rabiei, Colletotrichum lindemuthianum*	PP	MG	[4]
Brassica juncea	*Fusarium graminearum*	PP	MG	[5]
Brassica juncea	*Fuarium oxysporum, Macrophomina phaseolina, Rhizoctonia solani, Sclerotium rolfsii*	PP	MG	[6]
Brassica juncea	*R. solani*	PP	MG	[7]
Brassica juncea and *Sinapis alba* mixture	*Sclerotinia sclerotiorum*	PP	MG	[8]
Brassica napus	*F. graminearum*	PP	MG	[5]
Brassica napus	*Alternaria brassicae, S. sclerotiorum*	PP	MG	[9]
Brassica napus	*Verticillium longisporum, Verticillium dahliae*	PP	MG	[10]
Brassica napus	*Rhizoctonia solani*	PP	MG	[11]
Brassica napus	*R. solani*	PP	MG	[7]
Brassica napus	*S. sclerotiorum*	PP	MG	[12]
Brassica napus	*S. sclerotiorum*	PP	MG	[13]
Brassica nigra	*S. sclerotiorum*	PP	MG	[13]
Brassica oleracea	*A. brassicae, S. sclerotiorum*	PP	MG	[9]
Brassica oleracea	*Paxillus involutus, Paxillus tinctorius*	ENV	growth stimulation	[14]
Brassica rapa	*A. brassicae, S. sclerotiorum*	PP	MG	[9]
Brassica rapa	*Aspergillus niger, Candida albicans, Fusarium oxysporum*	HR, PP	MG	[15]
Brassica rapa	*P. involutus, P. tinctorius*	ENV	growth stimulation	[14]
Brassica rapa	*A. niger, C. albicans, F. oxysporum*	HR, PP	MG	[16]
Brassica rapa	*R. solani*	PP	MG	[7]
Eruca sativa	*Acremonium kiliense, Alternaria alternata, Aspergillus nidulans, Curvularia clavata, Dreschlera halodes, F. oxysporum, Rhizopus oryzae*	PP	MG	[17]
Eruca sativa	*Paecilomyces variotii, Penicillium lilacinum, Penicillium funiculosum, Spadicoides stoveri*	ENV	MG	[18]
Sinapis alba	*S. sclerotiorum*	PP	MG	[13]

Abbreviations: ENV, environmental (decaying fungi, molds, etc.); HR, human related (pathogens, clinical isolates, dermatophytes, etc.); MG, mycelial growth; PP, plant pathogen.

References

1. Bhattacharya, S.; Mayland-Quellhorst, S.; Müller, C.; Mummenhoff, K. Two-Tier Morpho-Chemical Defence Tactic in Aethionema via Fruit Morph Plasticity and Glucosinolates Allocation in Diaspores. *Plant Cell and Environment* **2019**, *42*, 1381–1392, doi:10.1111/pce.13462.
2. Jain, N.; Sharma, M. Insignificant Antidermatophytic Activity of Brassica Campestris Oil. *Journal of Pharmaceutical Negative Results* **2014**, *5*, 22–24, doi:10.4103/0976-9234.136784.
3. Morales-Rodríguez, C.; Bastianelli, G.; Aleandri, M.; Chilosi, G.; Vannini, A. Application of Trichoderma Spp. Complex and Biofumigation to Control Damping-Off of Pinus Radiata D. Don Caused by Fusarium Circinatum Nirenberg and O'Donnell. *Forests* **2018**, *9*, 421, doi:10.3390/f9070421.
4. Pane, C.; Villecco, D.; Roscigno, G.; Falco, E.D.; Zaccardelli, M. Screening of Plant-Derived Antifungal Substances Useful for the Control of Seedborne Pathogens. *Archives of Phytopathology and Plant Protection* **2013**, *46*, 1533–1539, doi:10.1080/03235408.2013.771458.
5. Leplat, J.; Heraud, C.; Gautheron, E.; Mangin, P.; Falchetto, L.; Steinberg, C. Colonization Dynamic of Various Crop Residues by Fusarium Graminearum Monitored through Real-Time PCR Measurements. *Journal of Applied Microbiology* **2016**, *121*, 1394–1405, doi:10.1111/jam.13259.
6. Fayzalla, E.A.; El-Barougy, E.; El-Rayes, M.M. Control of Soil-Borne Pathogenic Fungi of Soybean by Biofumigation with Mustard Seed Meal. *Journal of Applied Sciences* **2009**, *9*, 2272–2279, doi:10.3923/jas.2009.2272.2279.
7. Ascencion, L.C.; Liang, W.-J.; Yen, T.-B. Control of Rhizoctonia Solani Damping-off Disease after Soil Amendment with Dry Tissues of Brassica Results from Increase in Actinomycetes Population. *Biological Control* **2015**, *82*, 21–30, doi:10.1016/j.biocontrol.2014.11.010.
8. Rahmanpour, S.; Backhouse, D.; Nonhebel, H.M. Reaction of Glucosinolate-Myrosinase Defence System in Brassica Plants to Pathogenicity Factor of Sclerotinia Sclerotiorum. *European Journal of Plant Pathology* **2010**, *128*, 429–433, doi:10.1007/s10658-010-9685-y.
9. Sotelo, T.; Lema, M.; Soengas, P.; Cartea, M.E.; Velasco, P. In Vitro Activity of Glucosinolates and Their Degradation Products against Brassica-Pathogenic Bacteria and Fungi. *Applied and Environmental Microbiology* **2015**, *81*, 432–440, doi:10.1128/AEM.03142-14.
10. Zhou, L.; Hu, Q.; Johansson, A.; Dixelius, C. Verticillium Longisporum and V. Dahliae: Infection and Disease in Brassica Napus. *Plant Pathology* **2006**, *55*, 137–144, doi:10.1111/j.1365-3059.2005.01311.x.
11. Asad-Uz-Zaman, M.; Bhuiyan, M.R.; Khan, M.A.I.; Bhuiyan, M.K.A.; Latif, M.A. Integrated Options for the Management of Black Root Rot of Strawberry Caused by Rhizoctonia Solani Kuhn. *Comptes Rendus - Biologies* **2015**, *338*, 112–120, doi:10.1016/j.crvi.2014.11.006.
12. Dandurand, L.-M.; Mosher, R.D.; Knudsen, G.R. Combined Effects of Brassica Napus Seed Meal and Trichoderma Harzianum on Two Soilborne Plant Pathogens. *Canadian Journal of Microbiology* **2000**, *46*, 1051–1057.
13. Rahmanpour, S.; Backhouse, D.; Nonhebel, H.M. Toxicity of Hydrolysis Volatile Products of Brassica Plants to Sclerotinia Sclerotiorum, in Vitro. *Archives of Phytopathology and Plant Protection* **2014**, *47*, 1860–1865, doi:10.1080/03235408.2013.860723.
14. Zeng, R.S.; Mallik, A.U.; Setliff, E. Growth Stimulation of Ectomycorrhizal Fungi by Root Exudates of Brassicaceae Plants: Role of Degraded Compounds of Indole Glucosinolates. *Journal of Chemical Ecology* **2003**, *29*, 1337–1355, doi:10.1023/A:1024257218558.
15. Chung, I.-M.; Rekha, K.; Rajakumar, G.; Thiruvengadam, M. Production of Glucosinolates, Phenolic Compounds and Associated Gene Expression Profiles of Hairy Root Cultures in Turnip (Brassica Rapa Ssp. Rapa). *3 Biotech* **2016**, *6*, doi:10.1007/s13205-016-0492-9.
16. Thiruvengadam, M.; Baskar, V.; Kim, S.-H.; Chung, I.-M. Effects of Abscisic Acid, Jasmonic Acid and Salicylic Acid on the Content of Phytochemicals and Their Gene Expression Profiles and Biological Activity in Turnip (Brassica Rapa Ssp. Rapa). *Plant Growth Regul* **2016**, *80*, 377–390, doi:10.1007/s10725-016-0178-7.
17. Ali, A.; Bashir, U.; Haider, M. Bio-Control Effect of Eruca Sativa Mill. Oil against the Hazardous Food Borne Pathogens. Available online: /paper/Bio-control-effect-of-Eruca-sativa-Mill.-oil-the-Ali-Bashir/c84bfadcaf8f2b1b33a584d6765b97f8f7084b88 (accessed on 16 June 2020).
18. Rani, I.; Akhund, S.; Suhail, M.; Abro, H. Antimicrobial Potential of Seed Extract of Eruca Sativa. *Pakistan Journal of Botany* **2010**, *42*, 2949–2953.
