# Supplementary material for: Effects of Glucosinolate-Derived Isothiocyanates on Fungi: A Comprehensive Review on Direct Effects, Mechanisms, Structure-Activity Relationship Data and Possible Agricultural Applications
Source: J Fungi (Basel). 2021 Jul 6;7(7):539. doi: 10.3390/jof7070539 (PMC8305656; doi:10.3390/jof7070539)
Supplement: Supplementary file 1 [file jof-07-00539-s001.zip › Supplementary_table_3.pdf]

Supplementary table 3. Studies including *in vitro*, pot and field experiments with ITCs or glucosinolate containing plant biofumigants.

Biofumigant	Plant model	Protection against	Ref.
**Pure ITCs**			
Allyl / benzyl / butyl / ethyl / methyl / phenethyl / phenyl ITC	*Capsicum annuum*	*Sclerotinia sclerotiorum*	[47]
Allyl / ethyl / methyl ITC	*Citrus reticulata*	*Geotrichum citri-aurantii*	[49]
Allyl ITC	*Solanum lycopersicum*	*Fusarium* spp.	[75]
Allyl ITC	*Brassica oleracea* / *Phaseolus vulgaris*	*Rhizoctonia solani*	[213]
**Glucosinolate containing plants**			
Brassica carinata	*Ocimum basilicum* / *Lactuca sativa*	*Fusarium oxysporum*	[156]
Brassica carinata	*Fragaria × ananassa*	*Verticillium dahliae*	[158]
Brassica carinata	*Pinus radiata*	*Fusarium circinatum*	[161]
Brassica carinata / *B. juncea* / *Raphanus sativus* / *Sinapis alba*	*Zea mays*	*Fusarium graminearum*	[113]
Brassica juncea	*Brassica oleracea*	*Rhizoctonia solani*	[104]
Brassica juncea	*Beta vulgaris* / *Triticum aestivum*	*Rhizoctonia solani* / *Gaeumannomyces graminis*	[154]
Brassica juncea	*Glycine max*	*Fusarium oxysporum* / *Macrophomina phaseolina* / *Rhizoctonia solani* / *Sclerotium rolfsii*	[155]
Brassica juncea	*Solanum torvum*	*Verticillium dahliae*	[156]
Brassica juncea	*Malus domestica*	*Rhizoctonia solani*	[157]
Brassica juncea	*Oryza sativa*	*Rhizoctonia solani*	[160]
Brassica juncea / *B. napus* / *Sinapis alba*	*Triticum aestivum*	*Rhizoctonia solani*	[159]
Brassica juncea / *Camelina sativa*	*In vitro*	*Phymatotrichopsis omnivora*	[74]
Brassica napus	*Pisum sativum*	*Aphanomyces euteiches*	[152]
Brassica nigra / *B. oleracea* / *Sinapis alba*	*Gossypium hirsutum*	*Fusarium oxysporum*	[153]
Raphanus sativus / *Sinapis alba*	*Lupinus albus* / *Phaseolus vulgaris*	*Pleiochaeta setosa*	[48]

Abbreviations: ITC – isothiocyanate.

References (numbering as seen in the manuscript)

47. Kurt, S.; Güneş, U.; Soylu, E.M. In Vitro and in Vivo Antifungal Activity of Synthetic Pure Isothiocyanates against

Sclerotinia Sclerotiorum. *Pest Management Science* **2011**, *67*, 869–875, doi:10.1002/ps.2126.
49. Kara, M.; Soylu, E.M. Assessment of Glucosinolate-Derived Isothiocyanates as Potential Natural Antifungal Compounds against Citrus Sour Rot Disease Agent Geotrichum Citri-Aurantii. *Journal of Phytopathology* **2020**, *168*, 279–289, doi:10.1111/jph.12889.
75. Ren, Z.; Li, Y.; Fang, W.; Yan, D.; Huang, B.; Zhu, J.; Wang, X.; Wang, X.; Wang, Q.; Guo, M.; et al. Evaluation of Allyl Isothiocyanate as a Soil Fumigant against Soil-Borne Diseases in Commercial Tomato (Lycopersicon Esculentum Mill.) Production in China. *Pest Management Science* **2018**, *74*, 2146–2155, doi:10.1002/ps.4911.
213. Dhingra, O.D.; Costa, M.L.N.; Silva Jr., G.J. Potential of Allyl Isothiocyanate to Control Rhizoctonia Solani Seedling Damping off and Seedling Blight in Transplant Production. *Journal of Phytopathology* **2004**, *152*, 352–357, doi:10.1111/j.1439-0434.2004.00855.x.
156. Garibaldi, A.; Gilardi, G.; Clematis, F.; Gullino, M.L.; Lazzeri, L.; Malaguti, L. Effect of Green Brassica Manure and Brassica Defatted Seed Meals in Combination with Grafting and Soil Solarization against Verticillium Wilt of Eggplant and Fusarium Wilt of Lettuce and Basil. *Acta Horticulturae* **2010**, *883*, 295–302, doi:10.17660/ActaHortic.2010.883.36.
158. Yohalem, D.; Passey, T. Amendment of Soils with Fresh and Post-Extraction Lavender (Lavandula Angustifolia) and Lavandin (Lavandula×intermedia) Reduce Inoculum of Verticillium Dahliae and Inhibit Wilt in Strawberry. *Applied Soil Ecology* **2011**, *49*, 187–196, doi:10.1016/j.apsoil.2011.05.006.
161. Morales-Rodríguez, C.; Bastianelli, G.; Aleandri, M.; Chilosi, G.; Vannini, A. Application of Trichoderma Spp. Complex and Biofumigation to Control Damping-Off of Pinus Radiata D. Don Caused by Fusarium Circinatum Nirenberg and O'Donnell. *Forests* **2018**, *9*, 421, doi:10.3390/f9070421.
113. Vandicke, J.; Visschere, K.D.; Deconinck, S.; Leenknecht, D.; Vermeir, P.; Audenaert, K.; Haesaert, G. Uncovering the Biofumigant Capacity of Allyl Isothiocyanate from Several Brassicaceae Crops against Fusarium Pathogens in Maize. *Journal of the Science of Food and Agriculture* **2020**, *100*, 5476–5486, doi:https://doi.org/10.1002/jsfa.10599.
104. Chung, W.C.; Huang, J.W.; Huang, H.C.; Jen, J.F. Effect of Ground Brassica Seed Meal on Control of Rhizoctonia Damping-off of Cabbage. *Canadian Journal of Plant Pathology* **2002**, *24*, 211–218, doi:10.1080/07060660309506998.
154. Motisi, N.; Montfort, F.; Doré, T.; Romillac, N.; Lucas, P. Duration of Control of Two Soilborne Pathogens Following Incorporation of Above- and below-Ground Residues of Brassica Juncea into Soil. *Plant Pathology* **2009**, *58*, 470–478, doi:10.1111/j.1365-3059.2008.02017.x.
155. Fayzalla, E.A.; El-Barougy, E.; El-Rayes, M.M. Control of Soil-Borne Pathogenic Fungi of Soybean by Biofumigation with Mustard Seed Meal. *Journal of Applied Sciences* **2009**, *9*, 2272–2279, doi:10.3923/jas.2009.2272.2279.
157. Mazzola, M.; Zhao, X. Brassica Juncea Seed Meal Particle Size Influences Chemistry but Not Soil Biology-Based Suppression of Individual Agents Inciting Apple Replant Disease. *Plant and Soil* **2010**, *337*, 313–324, doi:10.1007/s11104-010-0529-5.
160. Handiseni, M.; Zhou, X.-G.; Jo, Y.-K. Soil Amended with Brassica Juncea Plant Tissue Reduces Sclerotia Formation, Viability and Aggressiveness of Rhizoctonia Solani AG1-IA towards Rice. *Crop Protection* **2017**, *100*, 77–80, doi:10.1016/j.cropro.2017.06.009.
159. Handiseni, M.; Brown, J.; Zemetra, R.; Mazzola, M. Effect of Brassicaceae Seed Meals with Different Glucosinolate Profiles on Rhizoctonia Root Rot in Wheat. *Crop Protection* **2013**, *48*, 1–5.
74. Hu, P.; Wang, A.S.; Engledow, A.S.; Hollister, E.B.; Rothlisberger, K.L.; Matocha, J.E.; Zuberer, D.A.; Provin, T.L.; Hons, F.M.; Gentry, T.J. Inhibition of the Germination and Growth of Phymatotrichopsis Omnivora (Cotton Root Rot) by Oilseed Meals and Isothiocyanates. *Applied Soil Ecology* **2011**, *49*, 68–75, doi:10.1016/j.apsoil.2011.06.014.
152. Dandurand, L.-M.; Mosher, R.D.; Knudsen, G.R. Combined Effects of Brassica Napus Seed Meal and Trichoderma Harzianum on Two Soilborne Plant Pathogens. *Canadian Journal of Microbiology* **2000**, *46*, 1051–1057.
153. El-Refai, I.M. Suppression of Fusarium Oxysporum f. Sp. Vasinfectum by Volatiles Produced by Hydrolysis of Glucosinolates of Some Brassicaceae Species. *Bulletin of the Faculty of Science, Assiut University, D: Botany* **2003**, *32*, 121–131.
48. Dewitte, K.; Landschoot, S.; Carrette, J.; Audenaert, K.; Derycke, V.; Latré, J.; Vermeir, P.; Haesaert, G. The Potential of Brassicaceae Biofumigant Crops to Manage Pleiochaeta Setosa in Sustainable Lupin Cultivation. *Biological Control* **2019**, *132*, 161–168, doi:10.1016/j.biocontrol.2019.02.020.
