# Supplementary material for: Effects of Glucosinolate-Derived Isothiocyanates on Fungi: A Comprehensive Review on Direct Effects, Mechanisms, Structure-Activity Relationship Data and Possible Agricultural Applications
Source: J Fungi (Basel). 2021 Jul 6;7(7):539. doi: 10.3390/jof7070539 (PMC8305656; doi:10.3390/jof7070539)
Supplement: Supplementary file 1 [file jof-07-00539-s001.zip › Supplementary_table_4.pdf]

Supplementary table 4. Review of plant protection studies combining glucosinolate containing plant amendments and biocontrol bacterial or fungal strains

Biocontrol strain	Amendment	Plant model	Activity against	Ref.
Bacillus amyloliquefaciens	*Brassica carinata*	*Solanum lycopersicum, Phaseolus vulgaris, Lactuca sativa*	*F. oxysporum, R. solani, S. minor*	[201]
Trichoderma atroviride	*Brassica juncea*	*Daucus carota*	*R. solani*	[200]
Trichoderma harzianum	*Brassica alba, B. nigra, B. napus, B. rapa, B. juncea, B. carinata*	*in vitro*	*Fusarium oxysporum, Rhizoctonia solani, Sclerotium rolfsii, Sclerotinia sclerotiorum*	[169]
Trichoderma harzianum	*Brassica carinata*	*in vitro*	*Sclerotinia minor, S. sclerotium*	[199]
Trichoderma isolates	*Brassica carinata*	*Beta vulgaris*	*F. oxysporum, R. solani*	[198]

References (numbering as seen in the manuscript)

169. Prasad, P.; Kumar, J.; Pandey, S. Investigating Disease Controlling Ability of Brassica Volatiles and Their Compatibility with Trichoderma Harzianum. *Proc. Natl. Acad. Sci., India, Sect. B Biol. Sci.* **2018**, *88*, 887–896, doi:10.1007/s40011-016-0829-5.

198. Galletti, S.; Sala, E.; Leoni, O.; Burzi, P.L.; Cerato, C. Trichoderma Spp. Tolerance to Brassica Carinata Seed Meal for a Combined Use in Biofumigation. *Biological Control* **2008**, *45*, 319–327, doi:10.1016/j.biocontrol.2008.01.014.

199. Sanchi, S.; Odorizzi, S.; Lazzeri, L.; Marciano, P. Effect of Brassica Carinata Seed Meal Treatment on the Trichoderma Harzianum T39-Sclerotinia Species Interaction. *Acta Horticulturae* **2005**, *698*, 287–292, doi:10.17660/ActaHortic.2005.698.38.

200. Montfort, F.; Poggi, S.; Morlière, S.; Collin, F.; Lemarchand, E.; Bailey, D.J. Opportunities to Reduce Rhizoctonia Solani Expression on Carrots by Biofumigation with Indian Mustard. In Proceedings of the XXVIII International Horticultural Congress on Science and Horticulture for People (IHC2010): International Symposium on Plant 917; 2010; pp. 149–157.

201. Pane, C.; Villecco, D.; Zaccardelli, M. Combined Use of Brassica Carinata Seed Meal, Thyme Oil and a Bacillus Amyloliquefaciens Strain for Controlling Three Soil-Borne Fungal Plant Diseases. *Journal of Plant Pathology* **2017**, *99*, 77–84, doi:10.4454/jpp.v99i1.3798.
